# Computational-Driven Epitope Verification and Affinity Maturation of TLR4-Targeting Antibodies

**DOI:** 10.3390/ijms22115989

**Published:** 2021-06-01

**Authors:** Bilal Ahmad, Maria Batool, Moon-Suk Kim, Sangdun Choi

**Affiliations:** 1 Department of Molecular Science and Technology, Ajou University, Suwon 16499, Korea; bilalpharma77@gmail.com (B.A.); mariabatool.28@gmail.com (M.B.); moonskim@ajou.ac.kr (M.-S.K.); 2 S&K Therapeutics, Woncheon Hall 135, Ajou University, Suwon 16499, Korea

**Keywords:** antibody, epitope, molecular dynamics, mutation, toll-like receptor

## Abstract

Toll-like receptor (TLR) signaling plays a critical role in the induction and progression of autoimmune diseases such as rheumatoid arthritis, systemic lupus erythematous, experimental autoimmune encephalitis, type 1 diabetes mellitus and neurodegenerative diseases. Deciphering antigen recognition by antibodies provides insights and defines the mechanism of action into the progression of immune responses. Multiple strategies, including phage display and hybridoma technologies, have been used to enhance the affinity of antibodies for their respective epitopes. Here, we investigate the TLR4 antibody-binding epitope by computational-driven approach. We demonstrate that three important residues, i.e., Y328, N329, and K349 of TLR4 antibody binding epitope identified upon in silico mutagenesis, affect not only the interaction and binding affinity of antibody but also influence the structural integrity of TLR4. Furthermore, we predict a novel epitope at the TLR4-MD2 interface which can be targeted and explored for therapeutic antibodies and small molecules. This technique provides an in-depth insight into antibody–antigen interactions at the resolution and will be beneficial for the development of new monoclonal antibodies. Computational techniques, if coupled with experimental methods, will shorten the duration of rational design and development of antibody therapeutics.

## 1. Introduction

Toll-like receptors (TLRs), as featured pattern recognition receptors, are proven to operate as germline-encoded proteins recognizing conserved pathogen-associated molecular patterns [1]. Dysregulation of cellular activities by microbes or their products through TLR signaling affects both innate and adaptive immune responses [2]. Excessive activation of TLRs disrupts immune homeostasis and is triggered by persistent induction of proinflammatory cytokines and chemokines, thereby subsequently leading to the initiation of various inflammatory and autoimmune disorders such as systemic lupus erythematosus, sepsis, psoriasis, atherosclerosis and asthma [3]. TLR4, the first mammalian Toll protein characterized in humans [4], recognizes damage-associated molecular patterns in the debris released by injured tissues and necrotic cells as well as pathogen-associated molecular patterns, and is thus associated with the development of several acute and chronic disorders such as sepsis [5]. TLR4 in association with interleukin (IL) 29 plays a crucial role in synovial inflammation. IL-29 upregulates synovial-fibroblast TLR4, which enhances synovium inflammation in rheumatoid arthritis (RA). Elevated expression may be due to elevated numbers of macrophages that penetrate the synovium and promote RA [6,7]. Numerous studies have shown that TLR4 stimulates the expression of many proinflammatory cytokines that play a crucial part in myocardial inflammation, including myocarditis, myocardial infarction, ischemia-reperfusion, and heart failure [8]. Ample evidence confirms the participation of TLR4 in neuroinflammation, where activation of this receptor stimulates microglial expression and activation of NF-κB, as well as the induction of inflammatory cytokines IL-1β, IL-6 and tumor necrosis factor (TNF) α [9,10]. The involvement of TLR4 in the pathogenesis and aggravation of these diseases highlights its importance as a potential drug target. In the TLR family, TLR4 remains a priority drug target and has been extensively studied for its therapeutic potential in several inflammatory disorders; many of these therapeutics are undergoing clinical trials [11].

Antibody-based medicines are currently the most widely used form of biotherapeutics, and their market expanded rapidly in recent years with increasing numbers of such modalities receiving FDA approval. Compared to small-molecule drugs, monoclonal antibodies (mAbs) are considered more selective and highly effective and have become mainstream therapies for autoimmune and other hard-to-cure diseases. Moreover, they have revolutionized the treatment of cancers, where inflammation is regarded as a crucial factor [12]. Nonetheless, small-molecule drugs that lack specificity are likely to have off-target effects. These drugs can interact unexpectedly and undesirably with tissues, cells and cellular components. The application of small-molecule drugs has a greater number of adverse effects due to their lower specificity as compared to mAbs. On the contrary, the clinical approval rate is higher for mAbs, and their development is easier and considerably faster than sorganic small-molecule compounds.

The development of high-affinity mAbs for therapeutic purposes is still a holy grail of molecular engineering [13]. Researchers are using numerous empirical procedures, including site-directed mutagenesis, complementarity-determining region (CDR)-grafting, and phage display techniques to enhance the target-binding affinity of mAbs [14]. Computational approaches are coming onto the scene and facilitating the development and affinity enhancement of mAbs. Prior sequence and structural information on both antigens and mAbs, and their epitope and paratope insights, are of great value for enhancing the affinity and stability of mAbs. These computational methods involve structural bioinformatic techniques such as homology modeling, protein–protein docking, protein interface analysis and molecular dynamics (MD) for rational design of mAbs. They provide detailed insights into binding and unbinding mechanisms as well as structure kinetics of protein-protein, protein-ligand and antigen-antibody that can be used to guide ligand, protein, peptide and mAb design [15]. The modelling of mAb-antigen interaction, and applying attraction or repulsion filters for masking the nonparatope residues as in Cluspro program, substantially improves the antigen-antibody docking complexes [16]. Thus, false positive of an epitope can be removed. The computational techniques alone can be used for affinity maturation of selected mAbs. Techniques such as AbDesign, RosettaAntibodyDesign, OptCDR and OptMAVEn are categorized as ab initio protocols for the design of novel paratopes [17,18]. Machine learning and deep learning methods can help to design CDRs for human IgG antibodies with target affinity that is superior to that of candidates derived from phage display panning experiments [19,20]. Thus far, a few effective TLR4-targeting mAbs have been investigated in preclinical and clinical studies [21,22]. NI-0101, previously known as Hu 15C1, has been developed in BALB/c mice and proved to efficiently block the signaling of lipopolysaccharide-triggered TLR4 both in vitro and in vivo [22,23]. NI-0101 is a humanized immunoglobulin (Ig) G1κ mAb, which is engineered to interfere with the dimerization of TLR4 and to abrogate its downstream signaling. NI-0101 has been proved effective in lipopolysaccharide-treated healthy volunteers [24]; nonetheless, recent clinical findings suggest that to cure RA, inhibition of TLR4 alone is not sufficient [25]. A molecular understanding of antigen–antibody interaction and identification of their epitope–paratope hotspots are indispensable for affinity enhancement of known mAbs. Taking advantage of the epitope knowledge provided by Greg Elson’s group through alanine mutagenesis [22], Loyau et al. utilized structural, computational, and phage display techniques to enhance TLR4-binding affinity of Hu 15C1 and suggested a C2E3 derivative with better TLR4-binding affinity [26]. Overall, these studies predicted the TLR4-mAb binding interface through site-directed mutational analyses. Nevertheless, the conformational changes that occur in the TLR4 structure owing to these mutations have been overlooked. These mutation-driven conformational changes obstruct TLR4-mAb interaction. In this study, we used computational mutagenesis, molecular docking, MD simulations and molecular mechanics to delineate the dynamic binding interface of Hu 15C1 and its derivative C2E3 toward TLR4. The crucial data generated during this study regarding TLR4 epitopes will be helpful for designing efficient mAbs to block TLR4 signaling and to curb the associated immune complications.

## 2. Results

### 2.1. Computational TLR4 Epitope Mutagenesis and Network Analysis

**Figure 1:**
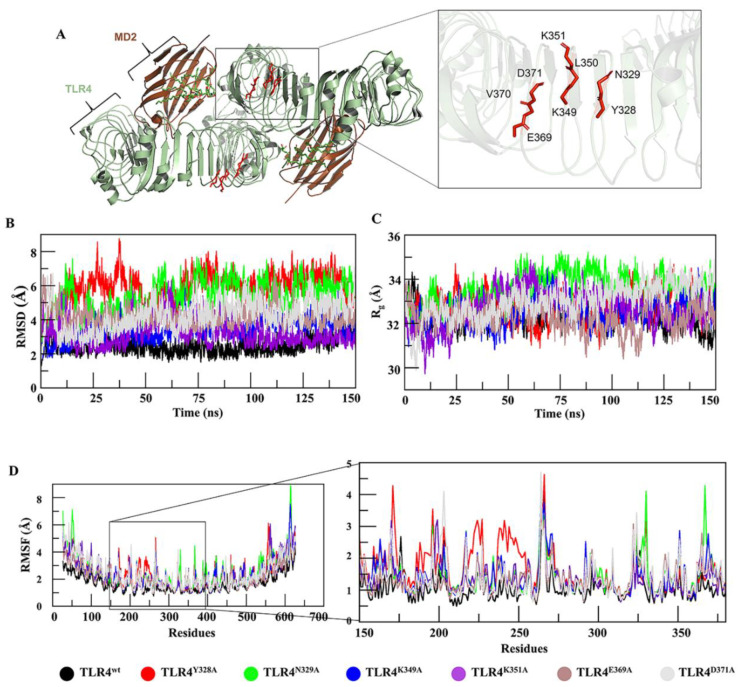
Characterization of an epitope using MD. (**A**) Epitope location in the TLR4–MD2 dimer interface and the residues of the epitope are labelled. (**B**) The mutational effect of these residues on TLR4 stability was analyzed by RMSD, where TLR4^Y328A^ (red), TLR4^N329A^ (green), and TLR4^D371A^ (grey) deviated substantially from TLR4^wt^ (black) while RMSD of TLR4^K349A^ (blue) remained constant. (**C**) *R_g_* determining the compactness of a protein was analyzed for TLR4^wt^ and its muteins. For TLR4^Y328A^ (red) and TLR4^N329A^ (green), the compactness was lost because their R*_g_* was higher than that of TLR4^wt^ (black). (**D**) RMSF of TLR4^wt^ and its muteins was measured. TLR4^N328A^, TLR4^Y329A^, and TLR4^K349A^ showed a fluctuation more vividly in the epitopic region as illustrated in the zoomed view.

Hu 15C1 is a potent anti-TLR4 mAb, which, owing to its efficacy, has reached clinical trials and is being evaluated in RA patients [25]. The epitope of mAb Hu 15C1 has been mapped by alanine mutagenesis and has been found to be located in the ectodomain of TLR4 near the dimerization interface [27]. This epitope has also been investigated in another study [26], in which a new derivative antibody, C2E3, was designed. By exploring the TLR4 structure, researchers hypothesize that the epitope-constituting residues suggested by these studies are crucial for preserving the structural integrity of the TLR4 backbone (Figure 1A). With the aim of gaining insights into TLR4 structure and mAb binding at the atomic level of specific residues, computational alanine scanning, and an MD simulation were performed here. Six hot spot residues, i.e., Y328, N329, K349, K351, E369 and D371, were individually mutated to alanine and vetted for changes in the interactions between TLR4 and a mAb.

Moreover, to comprehend the multiple interaction types involved in the residue network in wild-type TLR4 (TLR4^wt^) and its muteins, network analysis was performed. Substantial changes in the hydrogen bond network and in van der Waals interactions were observed at the mutation sites, and these changes led to a distinct conformational change in the epitope (Figure S1). Topological parameters of the residue interaction network (RIN) are given in Figure S1H. In the RIN of TLR4^wt^ and its muteins, there was a considerable variation in the node degree distribution. Thus, mutating these residues could distort the structure of TLR4 and make the precise Hu 15C1 epitope ambiguous.

### 2.2. MD of TLR4^wt^ and Muteins

Behaviors of TLR4 and its muteins were evaluated through conventional MD. Root mean square deviations (RMSDs) of Cα atoms of muteins and TLR4^wt^ were calculated as a function of time. Comparative RMSD plots suggested that TLR4^wt^ remained stable, while all the muteins underwent rigorous fluctuations (Figure 1B). The muteins manifested RMSDs in the following order: TLR4^Y328A^ > TLR4^N329A^ > TLR4^D371A^ > TLR4^K349A^ > TLR4^E369A^ > TLR4^K351A^ > TLR4^wt^. Additionally, to probe the effect of these mutations on TLR4 structure, root mean square fluctuation (RMSF) analysis was carried out, which confirmed that muteins TLR4^Y328A^, TLR4^N329A^, and TLR4^K349A^ underwent more fluctuations as compared to the other muteins and TLR4^wt^ structures (Figure 1D). Detailed analyses then suggested (discussed below) that these mutations affect the overall structure of TLR4, particularly the proposed epitope region. Owing to these mutation-driven substantial deviations in structural integrity, we concluded that these residues are more important for preserving TLR4 folding. Furthermore, the compactness of TLR4^wt^ and its muteins was determined by measuring the radius of gyration (*R_g_*). Differences in *R_g_* between TLR4^wt^ and muteins are presented in Figure 1C. TLR4^Y328A^, TLR4^N329A^ and TLR4^D371A^ showed significant increases in *R_g_*, indicating a decrease in the compactness of the system. In the second run of MD simulation the RMSD and *R_g_* (Figure S2) showed almost a similar trend except TLR4^K349A^ which underwent a bit higher RMSD fluctuation during 125–150 ns time-point (Figure S2D). To investigate the robustness of these results, an amber AMBER99SB-ILDN force field was implemented in all cases. All the trajectories showed a similar trend but with a lower RMSD than in the CHARMM36 force-field except TLR4^Y328A^ which showed fluctuation from the 65–85 ns time-point (Figure S2B). In compactness, a similar trend was followed with lower *R_g_* value as compared to the CHARMM36 force-field (Figure S2E,F). The two force-fields generally produce very similar overall RMSD and Rg. It has been found that CHARMM36 tends to produce more structure variation shifted towards disordered conformations, while as AMBER99SB-ILDN over stabilizes the native fold [28,29].

### 2.3. Mutation-Induced Conformational Changes in TLR4 Structure

**Figure 2:**
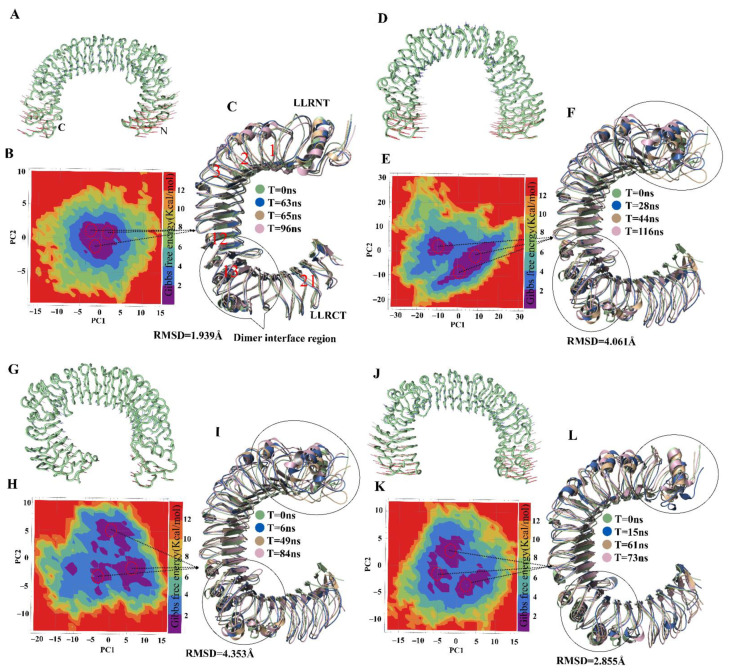
Motion and FEL of TLR4^wt^ and its muteins. The motion of N and C termini in a porcupine plot of (**A**) TLR4^wt^ is outward and in (**D**) it is inward with large magnitude, and for (**G**) TLR4^Y329A^ and (**J**) TLR4^K349A^, it is irregular. The FEL was computed using PC1 and PC2 as reaction coordinates for (**B**) TLR4^wt^, where all conformations remained confined to one minimum. (**E**) TLR4^Y328A^ conformations split into two minima with higher energy barrier. (**H**) TLR4^Y329A^ and (**K**) TLR4^K349A^ split into three minima. A representative lowest-energy structure from local minima indicated by red circles was superimposed (**C**,**F**,**I**,**L**) with the respective first or 0 ns frame structure in TLR4^wt^ and mutants shown along with the RMSD values. The black circle exemplifies the variation in structure at the epitopic region or dimer interface and termini.

To assess harmonic motions, NMA of TLR4^wt^ and muteins was performed. TLR4^wt^ showed an outward-motion tendency at both N and C termini; by contrast, TLR4^Y328A^ featured an inward movement at both termini (Figure 2A,B). The Y328A mutation seemingly changes the rigidity of the convex surface of TLR4 and allows the N and C termini to bend inward and come closer. A conformational change of such magnitude could affect the dimerization interface of TLR4 monomers in the (TLR4–MD2)^2^ complex (where MD2 is myeloid differentiation factor 2). The terminal regions of TLR4^N329A^ showed a vertical motion, inducing structural torsion, which resulted in a reduction in the number of internal hydrogen bonds and deformation of the solenoid structure of TLR4 (Figure 2C). TLR4^K349A^ underwent motions similar to those of TLR4^wt^ (Figure 2D). This finding was in line with the RMSF and RMSD plots, where TLR4^K349A^ did not show significant deviation and featured TLR4^wt^-like behavior. Other muteins, TLR4^K351A^, TLR4^E369A^ and TLR4^D371A^, showed a peculiar motion; however, the magnitude of these movements was not significant (Figure S3).

### 2.4. Visualization and Identification of Principal Motion of TLR4^wt^ and Muteins

The dominant motions in TLR4^wt^ and muteins were determined through principal component analysis (PCA) in which the first five eigenvectors captured most of MD motions. Most of the dynamic structural information for each system was captured by 5–6 eigenvectors with considerable fluctuations, while fluctuations of the remaining eigenvectors had low amplitude. The first three eigenvectors accounted for 63.9%, 67.4%, 75.2%, 63.6%, 75.0%, 65.2% and 74.7% of principal motions for TLR4^wt^, TLR4^Y328A^, TLR4^N329A^, TLR4^K349A^, TLR4^K351A^, TLR4^E369A^, and TLR4^D371A^, respectively (Figure S4). The first three eigenvectors of TLR4^wt^ and muteins were projected to determine interconformer relations (Figure S4). In Figure 2 and Figure S2, continuous color representation from red to blue indicates the change in conformation. The distribution of conformers in TLR4^wt^ remained mostly in two subspaces, i.e., red and blue. In the case of TLR4^Y328A^, the conformers were scattered into a subspace owing to periodic jumps between different conformations. Unlike TLR4^wt^, TLR4^N329A^ conformers dispersed and covered a large subspace, as was the case for TLR4^Y328A^. In the RMSD graph, TLR4^Y328A^ and TLR4^N329A^ showed instability because greater fluctuations were observed in PCA. The graph revealed continuous periodic jumps from one state to another, representing the instability of the alanine muteins of TLR4^Y328^ and TLR4^N329^. The transition between different conformations in these two muteins makes the epitope less exposed due to conformational changes. The other muteins showed different behavior in the PCA plots (Figure S4).

### 2.5. Energetics of Conformation Transitions

PCA was carried out to extract the predominant motions of the TLR4^wt^ and mutein structures; these data can provide a better picture of conformational changes and structural evolution along the trajectory of the MD simulation. The first two principal components were employed to calculate Gibbs free energy of each system via plotting in the free-energy landscape (FEL). A plateau in an FEL plot indicates the state of a protein corresponding to its energy and structural transition to the lowest-energy state along a simulation path. An overall view suggested that TLR4^wt^ remains in a single large global minima basin in the dark blue area (Figure 2B). Structure coordinates from the energy minima were randomly sampled to check conformational changes. Three samples were superimposed over the first frame. There was no substantial change in these structures, and their average RMSD was recorded and found to be 1.939 Å, which meant that TLR4^wt^ retains its conformation during the simulation.

In contrast, the energy plateau for TLR4^Y328A^, TLR4^N329A^ and TLR4^K349A^ was found to split into two, four and three energy minima, respectively, separated by high energy barriers (Figure 2E,H,K). Besides, the representative structures randomly sampled from these energy minima showed a significant change in their N and C termini. Moreover, conformational changes in leucine-rich repeat (LRR) regions were observed. LRR13 is in the proximity to the dimeric interface of TLR4 and manifested a significant difference in the muteins. The α-helix near the dimer interface also underwent substantial conformational changes. Nonetheless, TLR4^K351A^, TLR4^E369A^, and TLR4^D371A^ structures extracted at different time points from corresponding deep blue regions of the FEL did not show any difference in structural topology and RMSD (Figure S3).

### 2.6. The Influence of Mutations on the TLR4-mAb Interaction and Binding Affinity

**Figure 3:**
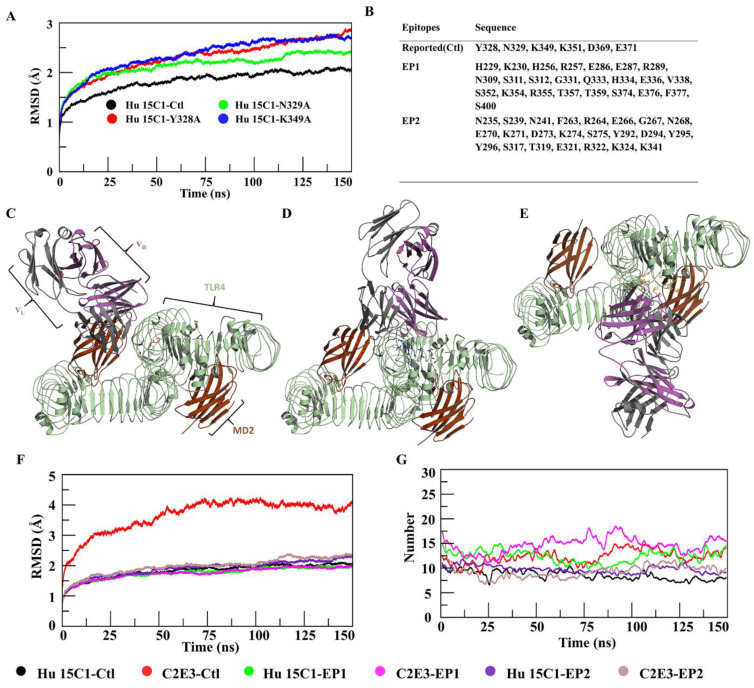
Epitope prediction and docking mode and stability analysis. (**A**) RMSDs of four complexes in which mutated complexes showed an increasing trend. (**B**) Residues for the construction of three conformational epitopes. Modes of docking of Hu 15C1 with TLR4 at sites (**C**) Ctl, (**D**) EP1, and (**E**) EP2 with superimposition of the human TLR4–MD2 dimer. RMSDs of six complexes are illustrated in (**F**), where C2E3–Ctl (red) showed a maximum deviation, and all remaining complexes were stable. The number of hydrogen bonds during the simulation was determined in (**G**), where C2E3–EP1 (magenta) featured the highest, C2E3–Ctl (red) and Hu 15C1–EP1 (green) moderate, and Hu 15C1–Ctl (black), Hu 15C1–EP2 (blue), and C2E3–EP2 (brown) the lowest number of hydrogen bonds formed during the simulation.

We noticed that the mutations caused various structural and conformational changes in TLR4, thereby increasing internal energy, and decreasing stability. Moreover, we noted that mutations TLR4^Y328A^, TLR4^N329^, and TLR4^K349A^, which mask the epitopes, influenced the structural integrity of TLR4. Taking into consideration the effect of these three mutations on TLR4, the mutated structures were docked with Hu 15C1 Fab and were simulated for 150 ns to gain insights into the detailed structural dynamics and comparative changes in binding affinity. Unlike the TLR4^wt^–Hu 15C1 complex, mutant complexes yielded a continuous rise in the RMSD plot (Figure 3A). The RMSD rise in these mutant complexes showed a similar trend when the AMBER99SB-ILDN force-field was implemented (Figure S5). This observation suggested that these site-specific point mutations led to structural changes in TLR4 that subsequently affect the binding and stability of the complex. Given that these mutations break the hydrogen bond network in the surroundings and partially mask epitope hotspots, we propose that these residues have an indirect impact on the binding to Hu 15C1.

To investigate the influence of these mutations on the binding affinity of TLR4 for Hu 15C1, binding free energies of TLR4–mAb complexes were calculated. As expected, we observed a decrease in the binding energy of mutein complexes as compared to the TLR4^wt^–Hu 15C1 complex (Table 1). Total binding free energy in each mutant system diminished by ~7–9%, which confirmed the decrease in the affinity of the mAb. The decrease in binding energy of the mutein complexes occurred because the interaction was reduced when residues, i.e., Y328, N329, and K349, were mutated into alanine in mutein complexes. The mutated residues do not interact with the antibody as in TLR4^wt^–Hu 15C1, which entails that the energy contributed by the mutated residue is lost. Thus, the decrease in the total binding energy of the mutein complexes was observed, suggesting that the original amino acids in this position is energetically vital for antigen binding.

### 2.7. Epitope Prediction and Interaction Analysis of the Epitope–Paratope Interface for Rational Design of Antibodies

The hotspot residues, as mentioned above, affect the conformation of TLR4, thereby reducing mAb-binding affinity. Therefore, we further investigated TLR4 epitopes and remapped them using conformational CDR information on Hu 15C1 and its derivative antibody, C2E3. Conformational epitopes on TLR4 were predicted based on an antibody–antigen score. Among the identified epitopes, EP1 and EP2 were selected and compared with a control (referred as Ctl) site. The residues of these epitopes are listed in Figure 3B and the Ramachandran plot of Ctl, EP1, and EP2 is shown in Figure S6. Ctl residues overlap with a few residues of EP1. Ctl is located near the TLR4 dimeric interface, whereas EP2 is located at the TLR4-MD2 interface. Hu 15C1 and C2E3 Fabs were docked to EP1, EP2, and Ctl epitope patches. In each resultant complex, the docked pose of a representative member from the most populated cluster was selected, as depicted in Figure 3C–E and Figure S5A–F. Superposition of the docking complexes revealed overlapping of one of mAb’s chain at sites Ctl and EP1 (Figure S7G,H).

To assess the stability of the TLR4–mAb complexes, MD simulations were performed and then analyzed. Hu 15C1–Ctl, Hu 15C1–EP1 and C2E3–EP1 had a similar pattern of the RMSD of backbone atoms, indicating that these complexes are highly stable. Nevertheless, C2E3–EP2 yielded a deviation in the last 75 ns, whereas C2E3–Ctl manifested a continuous increase in RMSD throughout the trajectory (Figure 3F). The marked RMSD increase could be explained by the finding that C2E3 induces a surge in the energy of epitopic or CDR residues upon TLR4 binding (data not shown). A similar tendency was observed in the RMSF plots of these complexes (Figure S7I,J). Residues 200–410 of TLR4 and 150–200 of the antibody in TLR4–C2E3 underwent more fluctuation compared to similar residues in the other complexes. The robustness of these finds was investigated under the effect of the AMBER99SB-ILDN force-field. The RMSD graph (Figure S8) of all the complexes showed a similar trend except C2E3–Ctl, although with a continuous increase in RMSD but much lower than when simulated with CHARMM36 (Figure S8B). Again, the lower RMSD in AMBER99SB-ILDN could be due to the overstabilization of native conformation. Nevertheless, C2E3–EP1 showed a similar trend in RMSD but slightly higher than in the CHARMM36 force field (Figure S8D).

To investigate the antigen–antibody interaction, we performed a dynamic analysis of the interaction of Hu 15C1 and C2E3 with TLR4; this approach provides the basis for rational design of mAbs. Primarily, the number of hydrogen bonds between each mAb and TLR4 was determined with a cutoff of <5 Å (Figure 3G). In Hu 15C1–Ctl, Hu 15C1–EP1, and C2E3–EP1, the total number of hydrogen bonds was 12–15 and persisted during the simulation. By contrast, in Hu 15C1–EP2 and C2E3–EP2, the hydrogen bond number during the simulation was within the range of 7–12.

**Figure 4:**
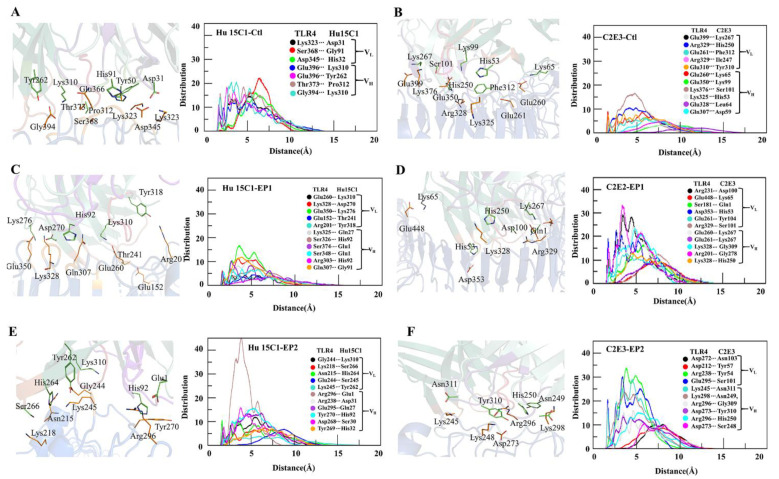
Distribution of minimum distances between the residues of Hu 15C1 and C2E3 interacting with TLR4 at sites (**A**,**B**) Ctl, (**C**,**D**) EP1, and (**E**,**F**) EP2.

To illustrate the distribution of interacting residues of CDRs in the antibodies toward TLR4, the radial distribution function and minimum distance were computed. In a comparison between the interactions of the two mAbs with the TLR4, the CDRs of the C2E3 antibody showed stronger affinity because the distribution of CDR residues interacting with epitopes was higher in comparison with Hu 15C1 (Figure 4). The molecular association of pairwise residue contacts within 5 Å toward TLR4 at three epitopic sites was greater for C2E3, indicating its stronger binding in comparison with Hu 15C1. The peak distribution of Hu 15C1 was higher at Ctl with a peak amplitude above 20 for the Ser368– Gly91 molecular association. The height of distribution peaks for Gly394–Lys310, Glu396–Asp310, and Thr373–Pro312 reached 20 and formed at shorter distances (Figure 4A). In comparison with Hu 15C1, the molecular association peaks formed for C2E3 toward TLR4 at Ctl had a lower amplitude. The pronounced peak of Lys376–Ser101 was above 15 and formed within the 5 Å distance. On the other hand, the rest of the peaks were smaller and formed at above 5 Å; therefore, the pairwise antibody–antigen residue contacts were weak (Figure 4B). The minimum distance for most of the interactions of the Hu 15C1–Ctl complex persisted during the simulation (Figure S9A and Table S1).

The minimum distance during the simulation in the C2E3–Ctl complex remained above 4 Å for some interacting residues (Figure S9B and Table S1). Both mAbs Hu 15C1 and C2E3 at the EP1 site were found to be buried inside, as evidenced by the docking pose (Figure 3C–E) where the maximum surface interacts with TLR4 at this site; therefore, a maximal interaction between the interacting residues was recorded. The highest peaks in the C2E3–EP1 complex formed at the shortest distance of <5 Å. The most pronounced peaks were rendered by Arg231–Asp100 and Arg201–Gly278 with an amplitude of 30, whereas the peaks formed by Asp353–His53, Glu260–Lys267, and Lys328–Gly278 were >20 in height (Figure 4D). In the Hu 15C1–EP1 complex, the molecular association peaks of Glu350–Lys278, Gln307–Gly91, and Lys328–Asp270 were above 10 and formed at a distance of <5 Å, while the amplitude of the rest of the contacts remained below 10 and formed at a distance of >5 Å (Figure 4C). The interacting residues in both complexes mostly remained intact except for Glu152–Thr241, Arg201–Tyr318, and Glu307–Gly91 in Hu 15C1–EP1 and Arg201–Ser278 in C2E3–EP1 during the simulation (Figure S9C,D and Table S2). The Hu 15C1 distribution of interactions remained below 10 except for the pronounced Arg296–Glu1 peak, which showed an amplitude up to 40 at the EP1 site. C2E3 featured a higher distribution of residue interactions at the EP2 site because Arg238–Tyr54, Glu295–Ser101 and Lys245–Asn311 peak amplitudes were approximately 30, and the peaks formed within the 5 Å distance (Figure 4E,F). In Hu 15 C1–EP2 and C2E3–EP2, the minimum distance between the interacting residues was registered above 5 Å for a few interactions (Figure S9E,F and Table S3). The radial distribution functions for Hu 15C1 and C2E3 at the EP1 site were more vigorous as the pronounced peaks formed at <5 Å distance, and the minimum distance of most of the interacting residues of Hu 15C1 and C2E3 at the EP2 site was >4 Å. In contrast to the EP1 site, the gap remained <4 Å during the simulation (Figure 4 and Figure S6). The interface interaction analysis revealed that at the EP1 site, most of the mAb–TLR4 interactions are energetically favorable, as suggested by the peak distribution at this epitope.

The interactions associated with the formation of paratope–epitope interface residues resulted in a free-energy change for the binding reaction. To compare the binding affinity for TLR4 between Hu 15C1 and C2E3 at epitopic sites Ctl, EP1 and EP2, total binding energies were computed by the molecular mechanics Poisson–Boltzmann surface area (MMPBSA) method. The total binding energies of complexes Hu 15C1–TLR4 and C2E3–TLR4 were −3358.261 and −5068.979 kJ/mol, respectively, at the EP1 site (Table 1), thus ensuring their stability in comparison to the Ctl site with a total binding energy of −2868.266 kJ/mol toward Hu 15C1 and −2797.202 kJ/mol toward C2E3. In Table 1, the binding free energies imply that the affinity of Hu 15C1 and C2E3 for the EP2 site is less than that for the EP1 site but greater than that for the control site. The binding free energy of both mAbs toward TLR4 at EP1 is much higher than that at EP2. The more negative the binding energy value, the stronger is the binding affinity of the antibody for its specific target antigen [30].

## 3. Discussion

Knowledge about a protein–protein interaction interface at the atomic level is essential for the development or design of substances intended to treat diseases. Similarly, the understanding of epitope–paratope interactions in an antibody–antigen complex at the atomic level is crucial for rational development of effective therapeutics.

An important step for the characterization of a functional antibody is accurate delineation of an epitope [31]. Insights about an epitope from a therapeutic perspective can be applied to rational design of mAbs [32]. On the other hand, a detailed epitope analysis is a major restraint because individual amino acid residues constituting an epitope are difficult to pinpoint. Given that experimental methods are time and resource consuming, and success is not guaranteed, computational techniques represent a rapid alternative for validation and characterization of an epitope and affinity maturation of mAbs [17].

In the present study, the epitope reported by the Elson group [22] was characterized and validated by in silico alanine mutagenesis. Epitopic residues in TLR4 were mutated to alanine to investigate the impact of each residue on TLR4 conformation and binding to an antibody. Among the six mutated residues, three, i.e., Y328, N329, and K349, were found to cause a conformational change in the TLR4 structure. RIN analysis suggested that these mutations induce a shift in residue topology owing to a loss of contact with the nearest neighbor or the formation of a new network in the protein structure (Figure S1). Because the epitope of TLR4 is conformational, the loss of the contact with the surrounding residues owing to a mutation will make the epitope ambiguous. The compactness of these muteins, as denoted by *R_g_*, showed a significant increase (Figure 2C). The higher *R_g_* values suggest that the mutations decreased structural stability. The MD simulation analysis of these mutations revealed the protein’s unstable behavior because RMSDs of the muteins uncovered variable behavior during the simulation, and residues from 175 to 400 (Hu 15C1–binding epitope) [26] of the mutated structures oscillated with a higher amplitude, as seen in the RMSF plot (Figure 1C). Gibbs FEL analysis indicated the energetic importance of these mutated residues for the stability of the TLR4 structure. The FEL plot contains splitting of the energy landscape with higher energy barriers, and similar trends were observed for all three mutations. The different conformations assumed by TLR4 owing to the mutations led to conformational changes in the epitopes and resulted in abrogation of the antibody binding, as illustrated by the PCA (Figure S4). The experimental data on the site-directed mutagenesis showed that one of these mutations, i.e., the K349 mutation, alters Hu 15C1 binding [26]. This result may be due to the conformational change in TLR4, which makes the Hu 15C1–binding epitope ambiguous. The impact of the mutation on TLR4 conformation, flexibility, and stability, as evidenced by NMA, showed that the protein folding energy may be increased and stability decreased. The motion of the termini of the muteins, causing twisting and deformity in the protein structure, leads to an increase in internal energy (Figure 2). Steric hindrance or torsion depending upon the direction of the motion induces an increase in the tension on intramolecular bonding, because of which internal hydrogen bonds between the residues break, which results in instability of the TLR4 solenoid structure. From thermodynamic and biophysical points of view, a conformational change in the TLR4 structure is due to the mutation and can make the conformational epitope ambiguous for Hu 15C1 binding. These observed impacts including steric clashes, hydrogen bond network disruption or abrogation of antibody binding, can lead to gain or loss in function of the receptor [33,34]. Moreover, Tsukamoto et al., assessed the functional role of TLR4 activation by a dual-luciferase NF-κB reporter assay, and demonstrated that the overexpression of TLR4^wt^ induced NF-κB activation was reduced upon transfection of the mutated plasmid in which LLR13 residues (TLR4 residing epitope) were mutated [27]. From these studies, we can assume that the mutation-driven structure change makes the antibody unable to recognize the epitope, and these residues play a functional role in TLR4 activation as they are essential for dimerization. We believe that the evidence is in support of our findings that mutations are responsible for structural change, which impacts the function of the protein [34,35].

To ascertain whether the mutations affected the binding affinity of Hu 15C1 and overall stability of the Hu 15C1–TLR4 complex, Hu 15C1 was docked with muteins TLR4^Y328A^, TLR4^N329A^ and TLR4^K349A^, and with TLR4^wt^. The backbone RMSD of the simulated mutein mAb-docked complexes uncovered an increasing deviation trend and consequently revealed the unstable nature of these mutein complexes (Figure 3A). Moreover, the binding-energy data showed a significant decline of binding affinity as compared to Ctl (Table 1). The hampering of mAb binding can be explained by the conformational changes in the TLR4 structure because of these mutations. The findings were next confirmed by PCA and FEL data, which suggested that the decrease in binding affinity may be due to the epitope-masking phenomenon. Several studies have shown that minimal structural changes in an antigen are sufficient to prevent antibody binding [36,37]. The effect of these mutations on binding affinity can influence the function of mAbs. Hu 15C1 did not manifest cross-reactivity in other species because of the ambiguity of the epitope owing to several mutations in TLR4 in these species [26]. Computationally assisted mutagenesis of an antigen or antibody, and a dynamic analysis of these muteins, are beneficial during antibody design for mutated epitopes and for maturation of mAbs. Recently, Takefumi et al. investigated a single-amino-acid substitution to improve antigen–antibody interaction by alanine scanning of interfacial residues. Furthermore, using MD, they found that the enthalpic improvement can be attributed to the stabilization of antigen–antibody interfaces [38]. The dynamics at atomic-level resolution of antigen–antibody interactions provide accurate insights for antibody development. To further investigate the allosteric binding of mAbs Hu 15C1 and C2E3 to TLR4, computationally assisted re-epitoping was performed. TLR4 is an important protein for the immune response. It may possess multiple binding sites because immune-response proteins have multiple and overlapping binding sites [39]. The Ctl epitope is located in the LLR13 region near the TLR4 dimeric interface [27]. One of the predicted epitopes, EP1, overlaps with Ctl. In our analysis of the paratope–epitope interaction interface for therapeutic and specificity purposes, Hu 15C1 and C2E3 were docked to two predicted epitopes. RMSD data on MD simulation trajectories of these complexes revealed that Hu 15C1 and C2E3 are highly stable and buried at EP1 (Figure 3C–F). In contrast to Ctl, this site provides a maximal surface for the interaction with mAbs Hu 15C1 and C2E3. The interface interaction analysis of Hu 15C1 and C2E3 at sites Ctl, EP1 and EP2 indicated that the molecular association of TLR4-mAb residues ensured the highest-peak formation at a shorter distance at the EP1 site, which means that these contacts are energetically preferred for both mAbs. Moreover, ionic and hydrogen bonds persisted during the simulation at the EP1 site, suggesting that Hu 15C1 and C2E3 are strongly bonded at the EP1 site in contrast to the other epitopic sites (Figure 4). The bonding of contacting residues is energetically favored because the distance between the interacting residues remains <4 Å during the simulation (Figure S9). The C2E3–Ctl interface interaction data showed an extended distance between the interacting residues during the simulation and energetically unfavorable interactions because the distribution peaks were short and formed at a distance of >5 Å, which could be the reason for the increased RMSD values. The binding-energy data support this finding. We observed the highest binding energy of Hu 15C1 at Ctl in contrast to the other epitopic sites; similarly, for C2E3, binding energy was the lowest. The salt bridge formation at the EP1 site for Hu 15C1 and C2E3 was greater, which provided extra stability to the complex. Salt bridge formation with a favorable geometrical orientation gives stability to the overall structure [40]. The binding energy data (Table 1) uncovered higher binding energy of C2E3 in comparison with Hu 15C1. It has been experimentally proved that C2E3 has stronger affinity than Hu 15C1 [41]. In short, the epitope analyses provided a comprehensive view of the TLR4 conformational changes that occur due to the mutations and indicated that they may yield epitope ambiguity. These outcomes will make the development of new mAbs easier and faster and will elucidate epitope structure for designing highly specific mAbs. For designing CDRs and their rationalization, the analyses of antibody–antigen interface interaction are important. Investigating the dynamic effect at the atomic level in the paratope–epitope interaction at three different TLR4 epitopic sites, i.e., Ctl, EP1, and EP2, for Hu 15C1 and C2E3, provided an in-depth understanding of these interactions. We believe that these findings will be helpful for rational design of high-affinity mAbs and for affinity maturation of already known mAbs Hu 15C1 and C2E3. Moreover, this study gives insights into a novel epitope at the TLR4-MD2 interface, indicating that heterodimerization of TLR4 and MD2 may be disrupted and that TLR4 signaling can be inhibited. In 2011, a group reported a MD2 mimicking peptide (MD2-I), which binds to the same site and disrupts the TLR4/MD2 interactions. MD2-I specificity has been evaluated in macrophages and animal models [42]. In addition to this, a few studies have also shown the importance of the druggability of this site [43,44,45].

mAbs have become an essential therapeutic tool, but their rationalization is a challenging phase and a crucial step in the development of highly specific mAbs. Comparative epitope analysis and antigen–antibody interface analysis via MD simulation offers an atomic-level understanding of the dynamic behavior of the antibody–antigen interactions, which is essential for antibody development. Furthermore, in comparison with other methods, the MMPBSA technique has an edge for the evaluation of affinity maturation and for clarification of the changes in binding affinity. However, TLR4 is a multidomain protein, but its full-length crystal structure is not available; therefore, we used the crystal structure of an isolated external domain in our study. The isolated domains can be less stable than the full-length multidomain protein but are fully functional [46]. In this study, we used computational methods for epitope verification and affinity maturation of TLR4-targeting antibodies. However, due to the limited facilities, we could not validate our findings experimentally, which can be performed in future studies. Therefore, for efficient mAb design, it is important to couple MD simulations with machine learning and deep learning methods. The structure from conformational space of the MD simulations can be utilized to find new plausible mAb conformations complementing pre-existing ones. We expect that the coupling of these computational methods with experimental methods will not only reduce the cost and time but also offer an efficient approach to the development of rationalized mAbs.

In this study, we demonstrated a computational-driven approach for the epitope verification and affinity maturation of TLR4 antibodies. By using network analysis and implicit MD simulation with FEL, we were able to determine the change in hydrogen bond network and van der Waals interactions in Y328, N329 and K349 among six mutated epitopic residues affecting structural integrity and the energy landscape of the TLR4 and, consequently, the antibody affinity. Further, we predicted the novel epitope located in the MD2 binding site region, which could be explored for new therapeutic antibodies. The uniqueness of our approach is that we successfully employed a wide array of computational-driven techniques to determine the dynamics of mAb-TLR4 interactions at the atomic level, and comparative epitope analysis by computing the binding affinity of the TLR4 antibodies. We believe that this computational-driven approach will accelerate the rational design of therapeutic antibodies. However, experimental validation of the present computational methods remains to be observed until more mAb-antigen are studied and experimentally verified. Moreover, coupling the present pipeline with deep learning and machine learning techniques can further enhance the feasibility of the method. Overall, this work shows that network analysis, MD simulation and MMPBSA techniques can be used as an exploratory methodology for the study of antibody rationalization.

## 4. Materials and Methods

### 4.1. In Silico Structure Reconstruction and Mutation of TLR4

The 3D coordinates of TLR4 (Protein Data Bank [PDB] ID: 3FXI) and TLR4-specific mAbs Hu 15C1 (PDB ID: 4R7D) and C2E3 (PDB ID: 4R7N) were retrieved from the RCSB PDB database. A monomeric TLR4 structure was first extracted from the TLR4–MD2 complex and optimized in Molecular Operating Environment (MOE) 2019.01. After the removal of water related to crystallization, hydrogen atoms and partial charges were added, incomplete and broken side chains were remodeled and hybridization events were adjusted at default structure preparation parameters in MOE. TLR4 muteins were created by the computational alanine-scanning method. The residues with low energy rotamers were selected and replaced with the amino acid alanine by means of a protein builder tool in the MOE suite. A similar protocol was used to generate TLR4–mAb complexes for the muteins followed by energy minimization to optimize the mutein structures.

### 4.2. Construction of the Interaction Network

Network description is widely used to analyze network topology and dynamics of complex systems. The RIN was constructed from a graph-based model of protein structure and topological residue connectivity, where residues are nodes and detected interactions are represented as edges. The RIN was constructed in RINalyzer with the help of the structure viz module implemented in Cytoscape 3.7.2 as per the protocol of Guilaume et al. [47]. The cutoff distance was set to <5 Å for residue interaction. The topological network parameters were calculated as an undirected network using a network analyzer [48].

### 4.3. Epitope Prediction and CDR Annotations

Conformational epitopes were predicted with the Epipred tool of the SAbPred web server [49,50]. Based on combined conformational matching of the antibody–antigen structures via geometric fitting and knowledge-based asymmetrical antibody–antigen scoring, epitopes of the antigen are listed rank-wise. The score of an epitope is given by:

(1) $$\mathrm{Epitope}\;\mathrm{Score}=\sum d\left(n\right)Pr\left(T_{\mathrm{ab}},\;T_{\mathrm{ag}}\right)$$

where ***T_ab_*** and ***T_ag_*** are the amino acid types of the antibody and antigen residues, respectively, that belong to node ***n***. The stereochemistry and geometry of selected epitopes was verified by Ramachandran plot via MOE’s inbuilt utility.

CDRs of antibodies retrieved from the PDB database are annotated according to the numbering scheme of Chothia and Lesk [51,52]. Instead of whole Fab regions, CDRs of the mAbs were considered ligand sites in the docking and simulation.

### 4.4. Molecular Docking and MD Simulation

Molecular docking of Hu 15C1 and C2E3 Fabs to TLR4 was performed on the Haddock 2.2 web server on the basis of the information derived from experimental and bioinformatics data [53]. The two mAbs were docked with the two newly predicted epitopes (discussed above) and a previously reported epitope (Ctl). In all three cases, the docked solutions generated by the Haddock server were clustered by their RMSDs. The RMSD measures the average distance of backbone atoms of the superimposed docked solutions, signifying the proximity between solutions. The most populated cluster with a lower RMSD was selected, and solutions with the lowest RMSD within the selected cluster were chosen for the MD analysis. The docking results obtained via Haddock were next validated by means of an antigen–antibody docking package implemented in the MOE suite.

MD simulations capture the dynamic behavior of proteins and other biomolecules in full atomic detail and provide an accurate insight into antigen-antibody interactions at very fine temporal resolution. The MD simulations were performed in GROMACS 2019.3 [54] on Intel E5-2680 and Intel E3-1275 with an Nvidia GeForce GTX 1060 graphics processing unit. TLR4, its muteins, and mAb–TLR4 complexes were solvated using the TIP3P water model in a cubic box; the dimensions of the box boundaries were extended by 10 Å from protein atoms. Na^+^ and Cl^−^ counter ions were added to neutralize the charge of the simulation system and energy minimization was performed using the CHARMM36 (Chemistry at Harvard macromolecular mechanics) and AMBER99SB-ILDN force-fields and a steep-descent algorithm [55]. V-rescale and Berendsen coupling schemes were employed for temperature and pressure equilibration procedures, respectively. After the temperature and pressure equilibration, MD simulations were carried out for 150 ns for each system in the CHARMM36 and AMBER99SB-ILDN force-fields.

### 4.5. Binding-Free-Energy Calculations

The MMPBSA approach was used to analyze free-energy interactions between the mAbs and TLR4. The enthalpy of the system was computed via molecular mechanics of MMPBSA, whereas the effect of the polar and nonpolar part of the solvent effect on free energy was determined via the Poisson–Boltzmann equation and calculation of the surface area. The basic equation is

(2) $$\Delta G_{\mathrm{bind}}=\Delta E_{\mathrm{MM}}+\Delta \Delta G_{\mathrm{sol}}-T\Delta S$$

where **Δ*G_bind_*** is binding free energy, **Δ*E_MM_*** represents the intramolecular energy difference in a vacuum, **ΔΔ*G_sol_*** is the solvation energy difference, ***T*** denotes absolute temperature and **Δ*S*** is the entropy change. In GROMACS, the built-in g_mmpbsa tool and APBS were called for the MMPBSA calculations, and the last 50 ns of an MD simulation trajectory of each complex with 10-frame intervals was extracted for the energy calculations. For the g_mmpbsa run, the dielectric constant of the aqueous solvent was set to 80, and the interior dielectric constant was set to 4; the surface tension constant g was set to 0.022 kJ/mol.

### 4.6. Principal Component Analysis and Free-Energy Landscape

PCA reduces a multidimensional dataset with many variables to a few ‘principal components’ that still preserve most of the differences between the data. In MD, PCA helps to elucidate the essential dynamics of the system in a low-dimensional FEL. PCA ultimately provides a view of the atoms (in the MD simulations) that move anisotropically to maximize the variance. To assess conformational flexibility of TLR4 and its muteins, PCA was performed in GROMACS, and the results were analyzed by means of the bio3D R package [56]. Before the PCA, translational and rotational motions were eliminated from the MD trajectory of all systems toward the average geometric center of the protein by least-square fit superimposition onto a reference structure [57]. To generate a covariance matrix, configurational space is reconstructed using a simple linear transformation in Cartesian coordinate space. Covariance matrix diagonalization generates an eigenvector set, where its vector components describe the direction of the motion, and the corresponding eigenvalue represents the magnitude of its energetic contribution to the motion. The FEL characterizes the state of a protein by exploring its conformations via an MD sampling technique and finds where Gibbs free energy for a sample is minimal. The gmx_sham tool implemented in GROMACS was used to predict the FEL through plotting of principal components PC1 and PC2 in the academic version of the Mathematica software (version 11.3; Wolfram Research, Inc., Champaign, IL, USA).

The DynaMut [58] machine-learning–based web server was employed to predict the impact of the mutations on TLR4 structure stability and flexibility. This tool predicts the harmonic motion of the wild-type and mutant TLR4 systems. By means of normal mode analysis (NMA), the nontrivial mode of a molecular motion of TLR4 and its mutants was studied and its vector representation was visualized in Pymol 2.3.

## Figures and Tables

**Table 1:** Binding free energy of Hu 15C1 and C2E3 toward TLR4.

Complex-Type	Vdw Energy	Electrostatic Energy	Polar Solvation	SASA	Binding Energy
Hu 15C1-Ctl	−379.741 +/− 18.392	−3222.642 +/− 100.808	776.278 +/− 54.509	−42.161 +/− 3.383	−2868.266 +/− 98.367
C2E3-Ctl	−458.220 +/− 22.391	−3339.736 +/− 98.973	1059.671 +/− 65.271	−58.917 +/− 4.214	−2797.202 +/− 97.581
Hu 15C1-Y328A	−341.401 +/− 20.569	−3232.006 +/− 172.050	1139.37 +/− 100.126	−56.347 +/− 3.599	−2490.384 +/− 169.412
Hu 15C1-N329A	−330.692 +/− 20.298	−3360.506 +/− 84.052	1377.683 +/− 124.152	−63.289 +/− 3.401	−2376.804 +/− 131.607
Hu 15C1-K349A	−336.232 +/− 20.298	−3260.506 +/− 84.052	1377.673 +/− 124.152	−55.389 +/− 3.401	−2274.454 +/− 131.607
Hu 15C1-EP1	−514.827 +/− 27.258	−4178.843 +/− 126.407	1398.977 +/− 85.365	−63.568 +/− 3.580	−3358.261 +/− 96.088
C2E3-EP1	−619.706 +/− 28.893	−6359.128 +/− 190.555	1996.806 +/− 109.523	−86.952 +/− 3.889	−5068.979 +/− 115.771
Hu 15C1-EP2	−385.218 +/− 19.617	−2301.460 +/− 71.852	1004.632 +/− 113.944	−48.778 +/− 3.287	−1730.824 +/− 122.321
C2E3-EP2	−344.892 +/− 19.082	−3348.181 +/− 107.983	885.182 +/− 71.196	−41.990 +/− 3.310	−2849.881 +/− 61.910

## Data Availability

The structure models and simulation trajectories are available upon request (sangdunchoi@ajou.ac.kr). All remaining data are contained within the manuscript.

## Supplementary Materials

Supplementary materials can be found at https://www.mdpi.com/article/10.3390/ijms22115989/s1.

## References

[1] Takeuchi O., Akira S. (2010). Pattern recognition receptors and inflammation. Cell.

[2] O’Neill L.A., Bryant C.E., Doyle S.L. (2009). Therapeutic targeting of Toll-like receptors for infectious and inflammatory diseases and cancer. Pharmacol. Rev..

[3] Shah M., Kim G.Y., Achek A., Cho E.Y., Baek W.Y., Choi Y.S., Lee W.H., Kim D.J., Lee S.H., Kim W. (2020). The alphaC helix of TIRAP holds therapeutic potential in TLR-mediated autoimmune diseases. Biomaterials.

[4] Medzhitov R., Preston-Hurlburt P., Janeway C.A. (1997). A human homologue of the Drosophila Toll protein signals activation of adaptive immunity. Nature.

[5] Singer M., Deutschman C.S., Seymour C.W., Shankar-Hari M., Annane D., Bauer M., Bellomo R., Bernard G.R., Chiche J.D., Coopersmith C.M. (2016). The Third International Consensus Definitions for Sepsis and Septic Shock (Sepsis-3). JAMA.

[6] Xu D., Yan S., Wang H., Gu B., Sun K., Yang X., Sun B., Wang X. (2015). IL-29 Enhances LPS/TLR4-Mediated Inflammation in Rheumatoid Arthritis. Cell. Physiol. Biochem..

[7] Achek A., Shah M., Seo J.Y., Kwon H.K., Gui X., Shin H.J., Cho E.Y., Lee B.S., Kim D.J., Lee S.H. (2019). Linear and Rationally Designed Stapled Peptides Abrogate TLR4 Pathway and Relieve Inflammatory Symptoms in Rheumatoid Arthritis Rat Model. J. Med. Chem..

[8] Yang Y., Lv J., Jiang S., Ma Z., Wang D., Hu W., Deng C., Fan C., Di S., Sun Y. (2016). The emerging role of Toll-like receptor 4 in myocardial inflammation. Cell Death Dis..

[9] Yao L., Kan E.M., Lu J., Hao A., Dheen S.T., Kaur C., Ling E.A. (2013). Toll-like receptor 4 mediates microglial activation and production of inflammatory mediators in neonatal rat brain following hypoxia: Role of TLR4 in hypoxic microglia. J. Neuroinflammation.

[10] Teng W., Wang L., Xue W., Guan C. (2009). Activation of TLR4-mediated NFkappaB signaling in hemorrhagic brain in rats. Mediat. Inflamm..

[11] Anwar M.A., Shah M., Kim J., Choi S. (2019). Recent clinical trends in Toll-like receptor targeting therapeutics. Med. Res. Rev..

[12] Haabeth O.A., Bogen B., Corthay A. (2012). A model for cancer-suppressive inflammation. Oncoimmunology.

[13] Beck A., Wurch T., Bailly C., Corvaia N. (2010). Strategies and challenges for the next generation of therapeutic antibodies. Nat. Rev. Immunol..

[14] Lu R.M., Hwang Y.C., Liu I.J., Lee C.C., Tsai H.Z., Li H.J., Wu H.C. (2020). Development of therapeutic antibodies for the treatment of diseases. J. Biomed. Sci..

[15] Bruce N.J., Ganotra G.K., Kokh D.B., Sadiq S.K., Wade R.C. (2018). New approaches for computing ligand-receptor binding kinetics. Curr. Opin. Struct. Biol..

[16] Kozakov D., Hall D.R., Xia B., Porter K.A., Padhorny D., Yueh C., Beglov D., Vajda S. (2017). The ClusPro web server for protein-protein docking. Nat. Protoc..

[17] Norman R.A., Ambrosetti F., Bonvin A., Colwell L.J., Kelm S., Kumar S., Krawczyk K. (2019). Computational approaches to therapeutic antibody design: Established methods and emerging trends. Brief. Bioinform..

[18] Yamashita T. (2018). Toward rational antibody design: Recent advancements in molecular dynamics simulations. Int. Immunol..

[19] Liu G., Zeng H., Mueller J., Carter B., Wang Z., Schilz J., Horny G., Birnbaum M.E., Ewert S., Gifford D.K. (2020). Antibody complementarity determining region design using high-capacity machine learning. Bioinformatics.

[20] Friedensohn S., Neumeier D., Khan T.A., Csepregi L., Parola C., de Vries A.R.G., Erlach L., Mason D.M., Reddy S.T. (2020). Convergent selection in antibody repertoires is revealed by deep learning. bioRxiv.

[21] Hatterer E., Shang L., Simonet P., Herren S., Daubeuf B., Teixeira S., Reilly J., Elson G., Nelson R., Gabay C. (2016). A specific anti-citrullinated protein antibody profile identifies a group of rheumatoid arthritis patients with a toll-like receptor 4-mediated disease. Arthritis Res. Ther..

[22] Dunn-Siegrist I., Leger O., Daubeuf B., Poitevin Y., Depis F., Herren S., Kosco-Vilbois M., Dean Y., Pugin J., Elson G. (2007). Pivotal involvement of Fcgamma receptor IIA in the neutralization of lipopolysaccharide signaling via a potent novel anti-TLR4 monoclonal antibody 15C1. J. Biol. Chem..

[23] Shang L., Daubeuf B., Triantafilou M., Olden R., Depis F., Raby A.C., Herren S., Dos Santos A., Malinge P., Dunn-Siegrist I. (2014). Selective antibody intervention of Toll-like receptor 4 activation through Fc gamma receptor tethering. J. Biol. Chem..

[24] Monnet E., Lapeyre G., Poelgeest E.V., Jacqmin P., Graaf K., Reijers J., Moerland M., Burggraaf J., Min C. (2017). Evidence of NI-0101 pharmacological activity, an anti-TLR4 antibody, in a randomized phase I dose escalation study in healthy volunteers receiving LPS. Clin. Pharmacol. Ther..

[25] Monnet E., Choy E.H., McInnes I., Kobakhidze T., de Graaf K., Jacqmin P., Lapeyre G., de Min C. (2020). Efficacy and safety of NI-0101, an anti-toll-like receptor 4 monoclonal antibody, in patients with rheumatoid arthritis after inadequate response to methotrexate: A phase II study. Ann. Rheum. Dis..

[26] Loyau J., Didelot G., Malinge P., Ravn U., Magistrelli G., Depoisier J.F., Pontini G., Poitevin Y., Kosco-Vilbois M., Fischer N. (2015). Robust Antibody-Antigen Complexes Prediction Generated by Combining Sequence Analyses, Mutagenesis, In Vitro Evolution, X-ray Crystallography and In Silico Docking. J. Mol. Biol..

[27] Tsukamoto H., Yamagata Y., Ukai I., Takeuchi S., Okubo M., Kobayashi Y., Kozakai S., Kubota K., Numasaki M., Kanemitsu Y. (2017). An inhibitory epitope of human Toll-like receptor 4 resides on leucine-rich repeat 13 and is recognized by a monoclonal antibody. FEBS Lett..

[28] Kamenik A.S., Handle P.H., Hofer F., Kahler U., Kraml J., Liedl K.R. (2020). Polarizable and non-polarizable force fields: Protein folding, unfolding, and misfolding. J. Chem. Phys..

[29] Jana K., Kepp K.P. (2020). Force-Field Benchmarking by Alternatives: A Systematic Study of Ten Small α- and β-Proteins. bioRxiv.

[30] Du X., Li Y., Xia Y.L., Ai S.M., Liang J., Sang P., Ji X.L., Liu S.Q. (2016). Insights into Protein-Ligand Interactions: Mechanisms, Models, and Methods. Int. J. Mol. Sci..

[31] Kringelum J.V., Lundegaard C., Lund O., Nielsen M. (2012). Reliable B cell epitope predictions: Impacts of method development and improved benchmarking. PLoS Comput. Biol..

[32] Kazi A., Chuah C., Majeed A.B.A., Leow C.H., Lim B.H., Leow C.Y. (2018). Current progress of immunoinformatics approach harnessed for cellular- and antibody-dependent vaccine design. Pathog. Glob. Health.

[33] Fowler D.M., Araya C.L., Fleishman S.J., Kellogg E.H., Stephany J.J., Baker D., Fields S. (2010). High-resolution mapping of protein sequence-function relationships. Nat. Methods.

[34] Studer R.A., Dessailly B.H., Orengo C.A. (2013). Residue mutations and their impact on protein structure and function: Detecting beneficial and pathogenic changes. Biochem. J..

[35] Schaefer C., Rost B. (2012). Predict impact of single amino acid change upon protein structure. BMC Genomics.

[36] Sela-Culang I., Kunik V., Ofran Y. (2013). The structural basis of antibody-antigen recognition. Front. Immunol..

[37] Chiu M.L., Goulet D.R., Teplyakov A., Gilliland G.L. (2019). Antibody Structure and Function: The Basis for Engineering Therapeutics. Antibodies.

[38] Yamashita T., Mizohata E., Nagatoishi S., Watanabe T., Nakakido M., Iwanari H., Mochizuki Y., Nakayama T., Kado Y., Yokota Y. (2019). Affinity Improvement of a Cancer-Targeted Antibody through Alanine-Induced Adjustment of Antigen-Antibody Interface. Structure.

[39] Zhao L., Wong L., Lu L., Hoi S.C., Li J. (2012). B-cell epitope prediction through a graph model. BMC Bioinform..

[40] Kumar S., Nussinov R. (2002). Close-range electrostatic interactions in proteins. Chembiochem.

[41] Loyau J., Malinge P., Daubeuf B., Shang L., Elson G., Kosco-Vilbois M., Fischer N., Rousseau F. (2014). Maximizing the potency of an anti-TLR4 monoclonal antibody by exploiting proximity to Fcgamma receptors. MAbs.

[42] Liu L., Ghosh N., Slivka P.F., Fiorini Z., Hutchinson M.R., Watkins L.R., Yin H. (2011). An MD2 hot-spot-mimicking peptide that suppresses TLR4-mediated inflammatory response in vitro and in vivo. Chembiochem.

[43] Nishitani C., Mitsuzawa H., Sano H., Shimizu T., Matsushima N., Kuroki Y. (2006). Toll-like receptor 4 region Glu24-Lys47 is a site for MD-2 binding: Importance of CYS29 and CYS40. J. Biol. Chem..

[44] Nishitani C., Mitsuzawa H., Hyakushima N., Sano H., Matsushima N., Kuroki Y. (2005). The Toll-like receptor 4 region Glu24-Pro34 is critical for interaction with MD-2. Biochem. Biophys. Res. Commun..

[45] Re F., Strominger J.L. (2003). Separate functional domains of human MD-2 mediate Toll-like receptor 4-binding and lipopolysaccharide responsiveness. J. Immunol..

[46] Bhaskara R.M., Srinivasan N. (2011). Stability of domain structures in multi-domain proteins. Sci. Rep..

[47] Brysbaert G., Mauri T., de Ruyck J., Lensink M.F. (2019). Identification of Key Residues in Proteins Through Centrality Analysis and Flexibility Prediction with RINspector. Curr. Protoc. Bioinform..

[48] Assenov Y., Ramirez F., Schelhorn S.E., Lengauer T., Albrecht M. (2008). Computing topological parameters of biological networks. Bioinformatics.

[49] Dunbar J., Krawczyk K., Leem J., Marks C., Nowak J., Regep C., Georges G., Kelm S., Popovic B., Deane C.M. (2016). SAbPred: A structure-based antibody prediction server. Nucleic Acids Res..

[50] Krawczyk K., Liu X., Baker T., Shi J., Deane C.M. (2014). Improving B-cell epitope prediction and its application to global antibody-antigen docking. Bioinformatics.

[51] Chothia C., Lesk A.M. (1987). Canonical structures for the hypervariable regions of immunoglobulins. J. Mol. Biol..

[52] Murzin A.G., Brenner S.E., Hubbard T., Chothia C. (1995). SCOP: A structural classification of proteins database for the investigation of sequences and structures. J. Mol. Biol..

[53] Ritleng P., Loubiere R., Marcelet B. (1989). [Suppurative pseudo-lithiasic canaliculitis]. Ophtalmologie.

[54] Abraham M.J., Murtola T., Schulz R., Páll S., Smith J.C., Hess B., Lindahl E. (2015). GROMACS: High performance molecular simulations through multi-level parallelism from laptops to supercomputers. SoftwareX.

[55] Huang J., Rauscher S., Nawrocki G., Ran T., Feig M., de Groot B.L., Grubmuller H., MacKerell A.D. (2017). CHARMM36m: An improved force field for folded and intrinsically disordered proteins. Nat. Methods.

[56] Skjaerven L., Yao X.Q., Scarabelli G., Grant B.J. (2014). Integrating protein structural dynamics and evolutionary analysis with Bio3D. BMC Bioinform..

[57] Amadei A., Linssen A.B., de Groot B.L., van Aalten D.M., Berendsen H.J. (1996). An efficient method for sampling the essential subspace of proteins. J. Biomol. Struct. Dyn..

[58] Rodrigues C.H., Pires D.E., Ascher D.B. (2018). DynaMut: Predicting the impact of mutations on protein conformation, flexibility and stability. Nucleic Acids Res..

