# Supplementary material for: Computational-Driven Epitope Verification and Affinity Maturation of TLR4-Targeting Antibodies
Source: Int J Mol Sci. 2021 Jun 1;22(11):5989. doi: 10.3390/ijms22115989 (PMC8198660; doi:10.3390/ijms22115989)
Supplement: Supplementary file 1 [file ijms-22-05989-s001.zip › ijms-1217440-supplementary.pdf]

Supplementary Material

Computational-Driven Epitope Verification and Affinity Maturation of TLR4-Targeting Antibodies

Bilal Ahmad [1], Maria Batool [1,2], Moon Suk Kim [1] and Sangdun Choi [1,2,*]

[1] Department of Molecular Science and Technology, Ajou University, Suwon 16499, Korea; bilalpharma77@gmail.com (B.A.); mariabatool.28@gmail.com (M.B.); moonskim@ajou.ac.kr (M.S.K.)
[2] S&K Therapeutics, Woncheon Hall 135, Ajou University, Suwon 16499, Korea
* Correspondence: sangdunchoi@ajou.ac.kr; Tel.: +82-31-219-2600; Fax: +82-31-219-1615

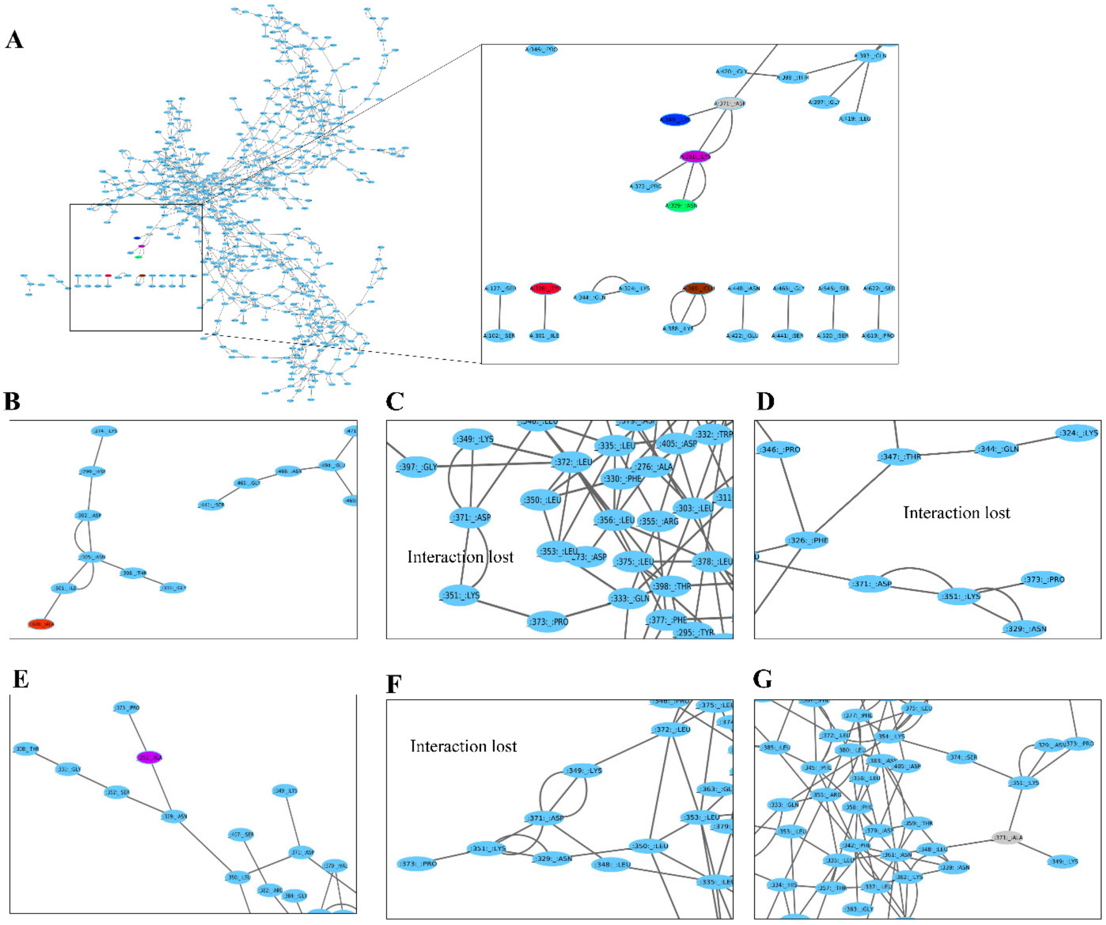

H

Network Parameters	WT	Y328A	N329A	K349A	K351A	E369A	D371A
Clustering coefficient	0.321	0.288	0.288	0.295	0.292	0.292	0.288
Network diameter	29	28	28	28	27	27	27
Network centralization	0.011	0.013	0.012	0.012	0.012	0.012	0.01
Characteristic path length	10.15	10.096	10.058	10.028	10.013	9.989	9.985
Avg. number of neighbors	6.173	6.03	6.113	6.023	6.013	5.93	5.937
Number of nodes	601	601	601	601	601	601	601
Network density	0.01	0.01	0.01	0.01	0.01	0.01	0.01
Network heterogeneity	0.374	0.374	0.376	0.368	0.379	0.372	0.365
Multi-edge node pairs	875	813	828	789	796	821	809
Number of edges	1041	1048	1090	1027	1038	1032	1018

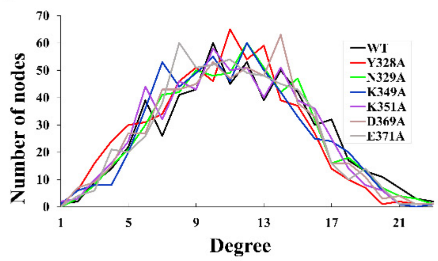

Figure S1. Network topology of the epitope of TLR4 and its muteins. (A) The residues constituting the epitope in the network are shown in red (Y328), green (N329), blue (K349), magenta (K351), brown (D369), and grey (E371). B) Y328A (red) forms a small community. The network interactions are lost in

(C) N329A, (D) K349A, and (E) D369A, whereas (B) Y328A forms a small community and (G) D371A remains a part of the whole network. (H) The residue network properties are shown in the table for the wild-type and mutant TLR4. (I) A node degree distribution is provided.

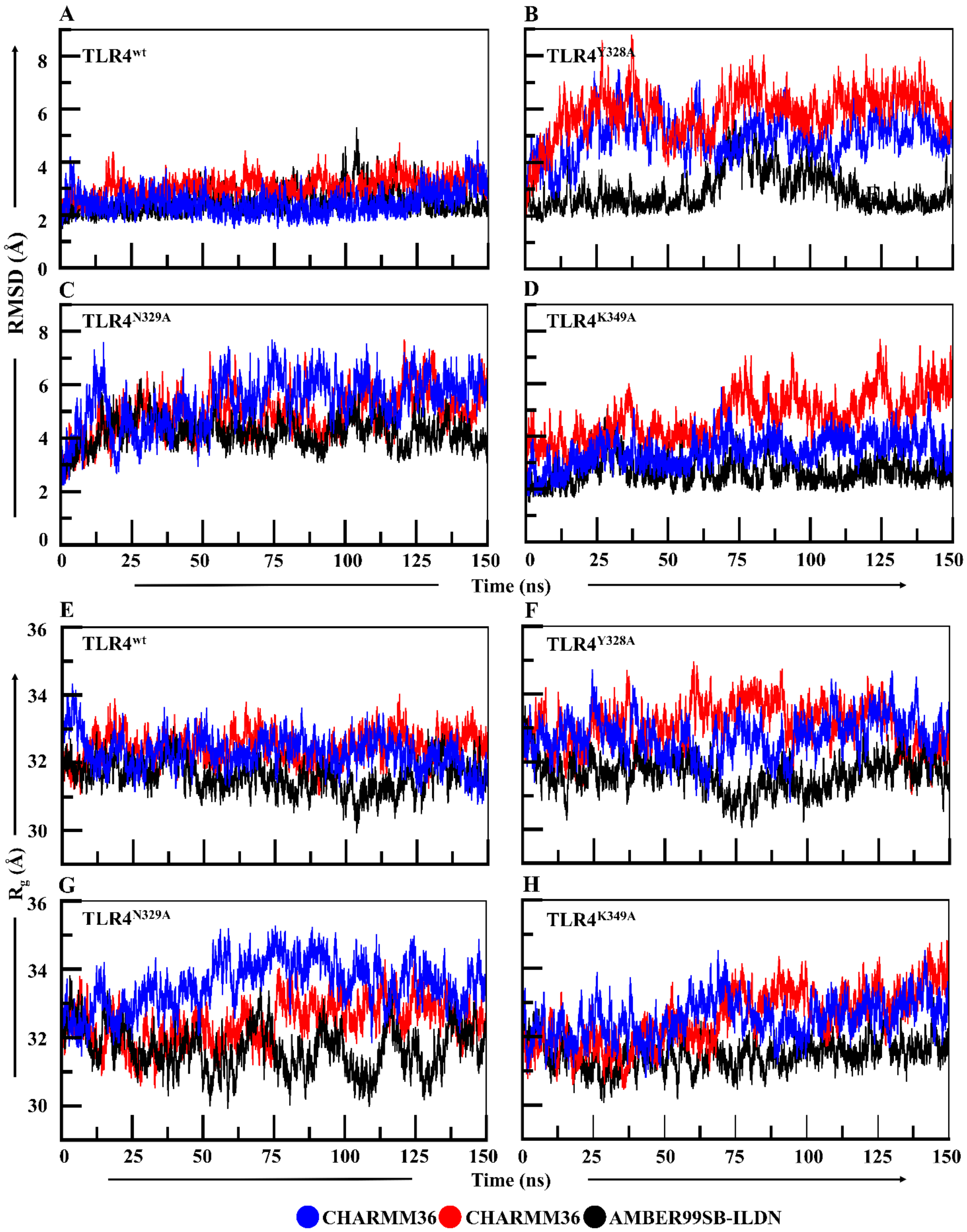

Figure S2. RMSD and R_g for TLR4^{wt} and muteins using CHARMM36 and AMBER99SB-ILDN force fields. The RMSD (A-D) of TLR4^{wt} and muteins showed a similar trend in both run of MD simulation when CHARMM36 (blue and red) force field was used except TLR4^{K349A} fluctuates a bit higher RMSD in 75-150ns time point. With the use of AMBER99SB-ILDN (black) force field RMSD was lower in all trajectories as compared to CHARMM36. R_g (E-H) of the TLR4 and muteins showed a similar pattern in both MD runs using CHARMM36 force field but R_g value is lower with AMBER99SB-ILDN force field.

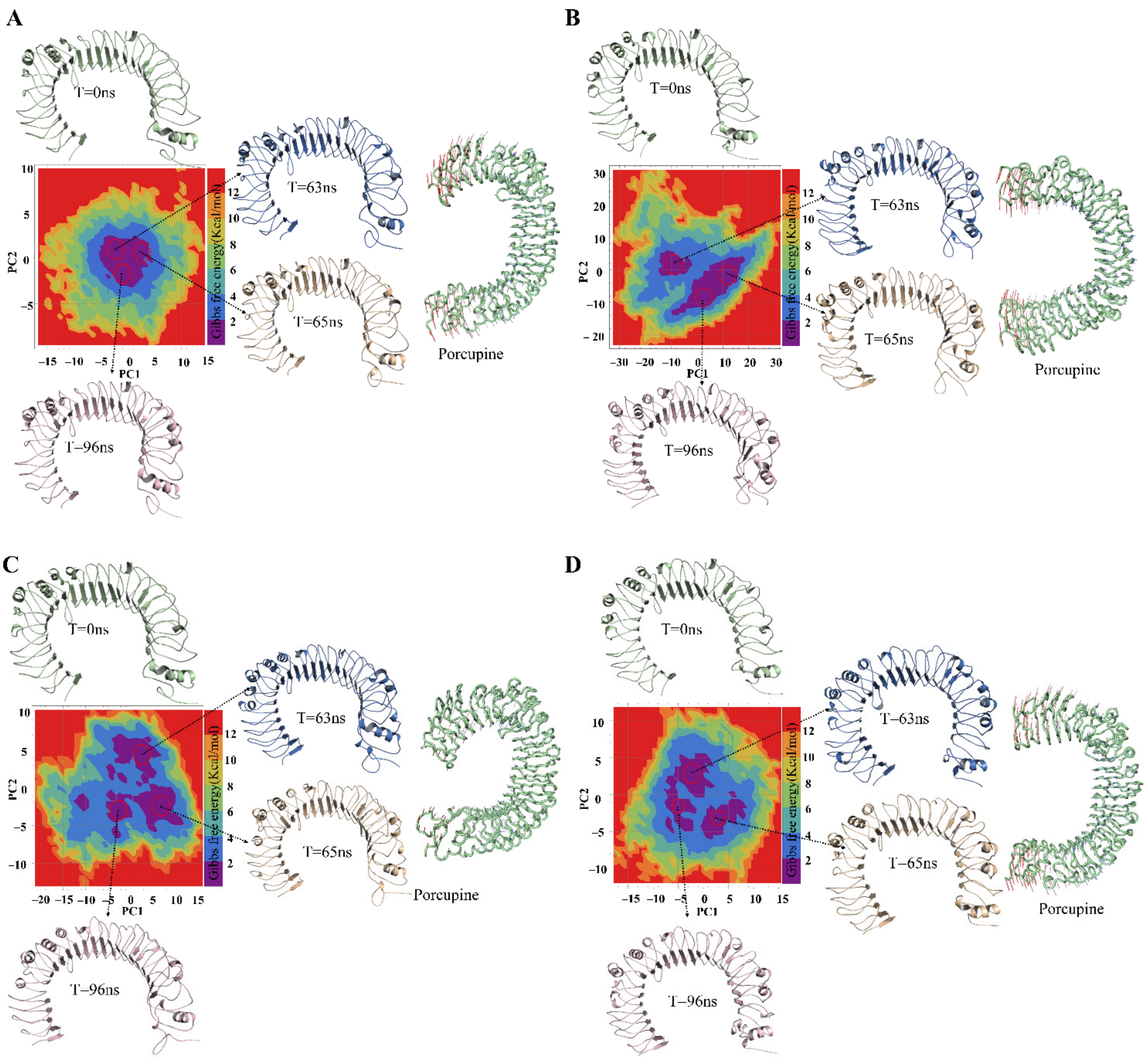

Figure S3. Continued…

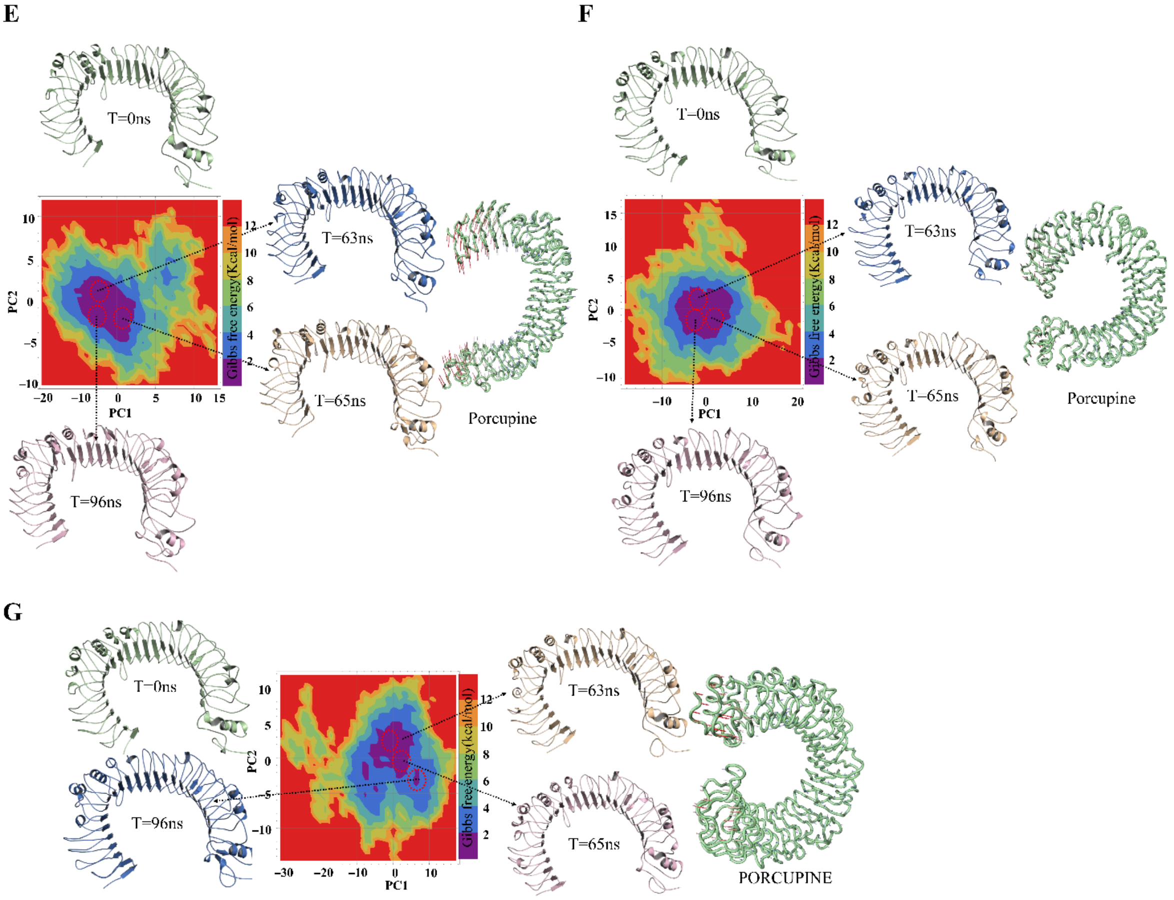

H

TLR4^{wt}

	T0	T1	T2	T3
T0	0.00	2.03	1.78	2.56
T1	2.03	0.00	1.47	1.76
T2	1.78	1.47	0.00	1.87
T3	2.56	1.76	1.87	0.00

TLR4^{Y328A}

	T0	T1	T2	T3
T0	0.00	5.31	4.81	5.47
T1	5.31	0.00	1.94	2.50
T2	4.81	1.94	0.00	2.75
T3	5.47	2.50	2.75	0.00

TLR4^{N329A}

	T0	T1	T2	T3
T0	0.00	4.72	4.90	5.97
T1	4.72	0.00	2.85	4.17
T2	4.90	2.85	0.00	2.51
T3	5.97	4.17	2.51	0.00

TLR4^{K349A}

	T0	T1	T2	T3
T0	0.00	2.73	2.85	3.41
T1	2.73	0.00	2.54	3.22
T2	2.85	2.54	0.00	2.22
T3	3.41	3.22	2.22	0.00

TLR4^{K351A}

	T0	T1	T2	T3
T0	0.00	2.42	2.89	2.68
T1	2.42	0.00	2.05	1.85
T2	2.89	2.05	0.00	2.32
T3	2.68	1.85	2.32	0.00

TLR4^{E369A}

	T0	T1	T2	T3
T0	0.00	3.75	3.56	3.62
T1	3.75	0.00	2.53	2.23
T2	3.56	2.53	0.00	1.74
T3	3.62	2.23	1.74	0.00

TLR4^{D371A}

	T0	T1	T2	T3
T0	0.00	3.99	3.73	4.52
T1	3.99	0.00	1.97	3.03
T2	3.73	1.97	0.00	2.87
T3	4.52	3.03	2.87	0.00

Figure S3. The FEL and representative structures. The FEL was computed from PC1 and PC2 as reaction coordinates for (A) TLR4^{wt}, E) TLR4^{K351A}, F) TLR4^{E369A}, and G) TLR4^{E369A} where all conformations remained confined to one minimum. (B) TLR4^{Y328A} conformations split into two minima with a higher energy barrier. (C) TLR4^{Y329A} and (D) TLR4^{K349A} split into three minima. A representative lowest-energy structure from local minima shown by red circles was extracted in (A, B, C, D, E, F, and G), and the porcupine plot represents the of motion of TLR4^{wt} and mutant. H) RMSDs with their respective first or 0 ns frame structure and with one another are shown.

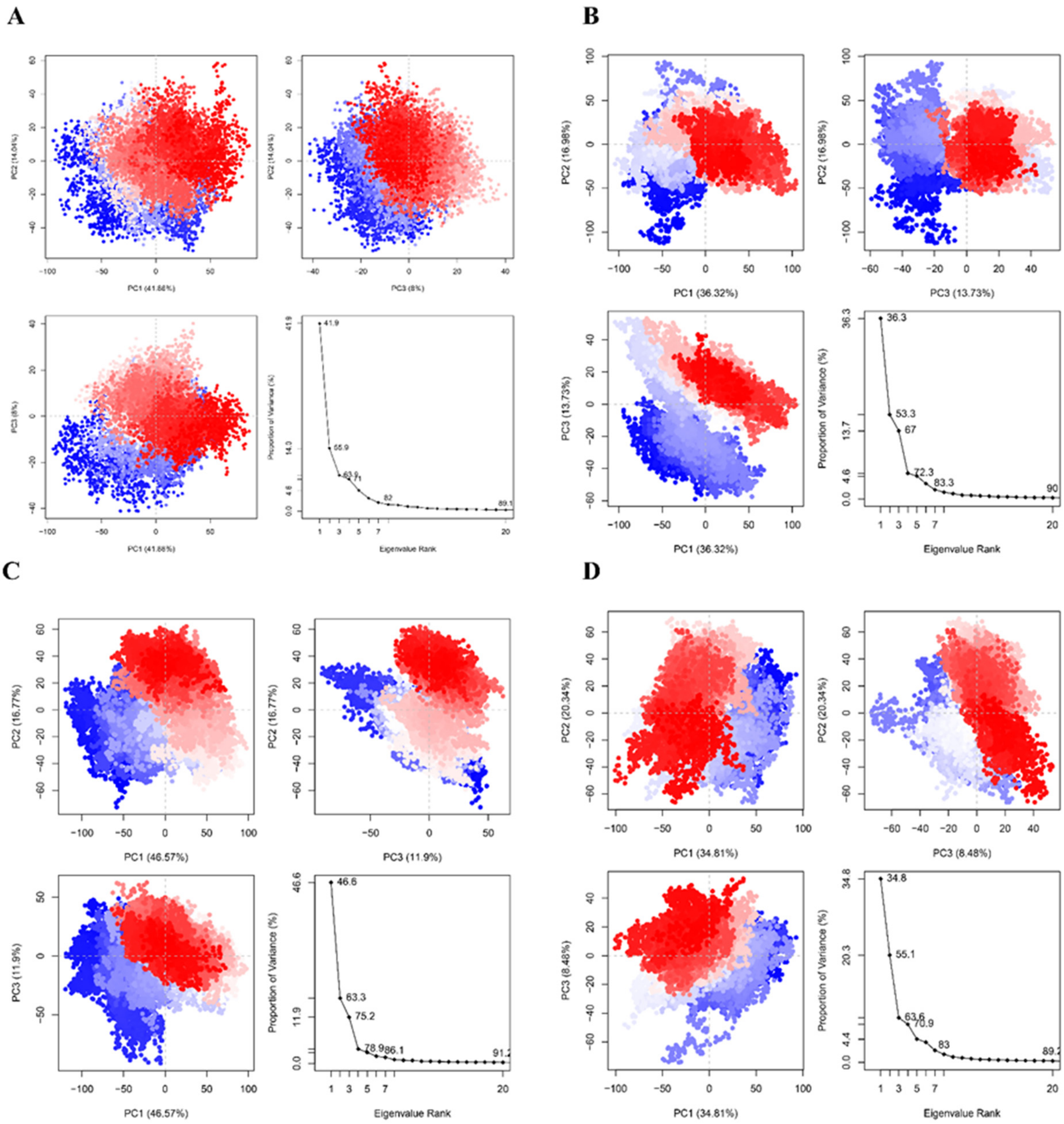

FigureS 4. Continued…

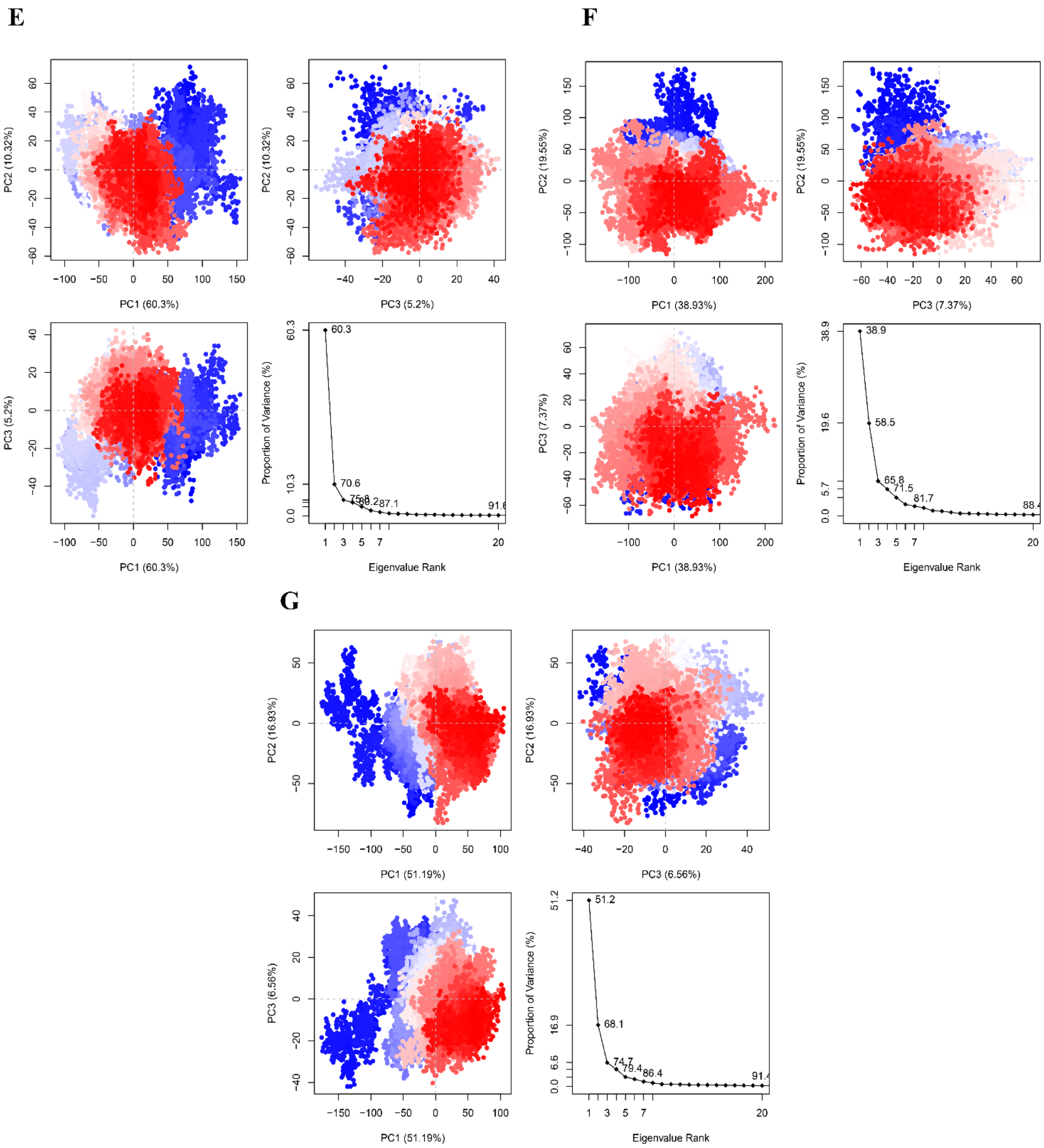

Figure S4. PCA for TLR4^{wt} and muteins. The cloud reflects (A) TLR4^{wt}, (B) TLR4^{Y328A}, (C) TLR4^{N329A}, (D) TLR4^{K349A}, (E) TLR4^{K351A}, (F) TLR4^{E369A}, and (G) TLR4^{D371A} trajectory projections. The total mean square displacement of residue positional fluctuations captured in each dimension is characterized by a corresponding eigenvalue (principal components; PCs). The continuous color scale from blue to red indicates that there are periodic jumps between these conformers throughout the trajectory.

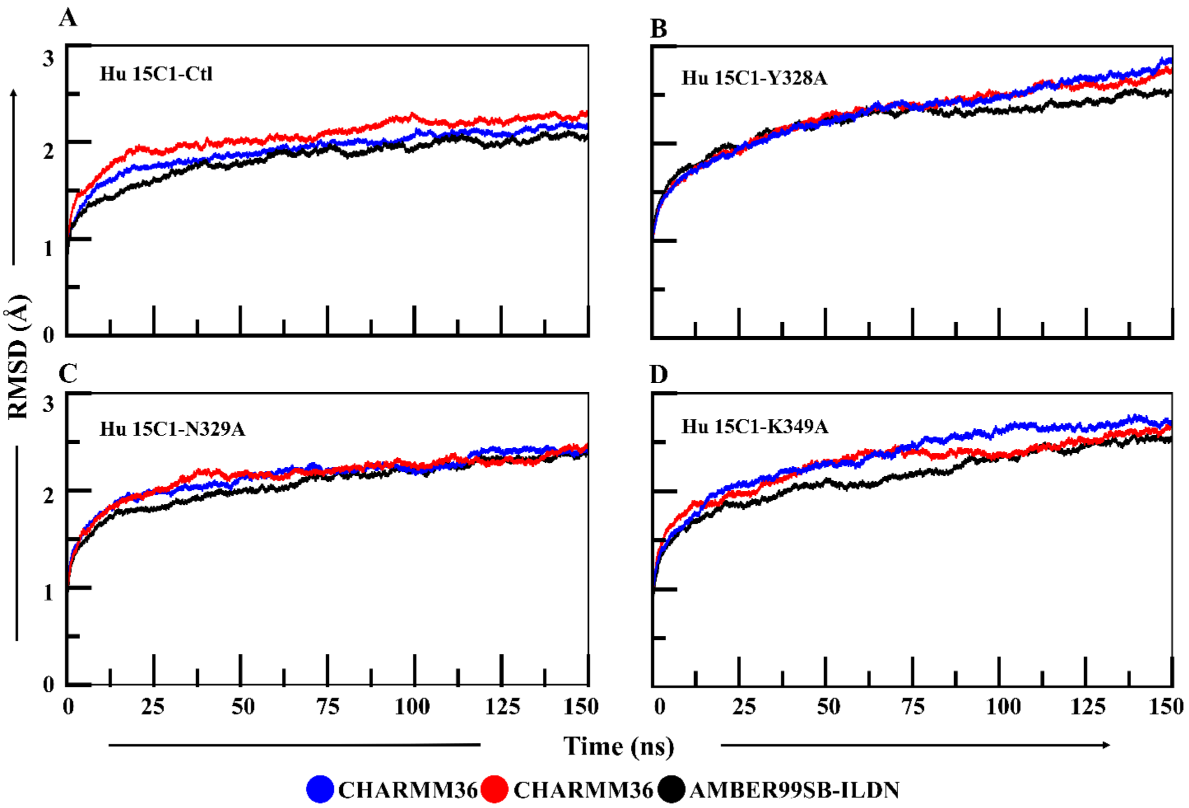

Figure S5. Effect of CHARMM36 (blue and red) and AMBER99SB-ILDN (black) force fields on RMSD of TLR4-mAb mutein complexes.

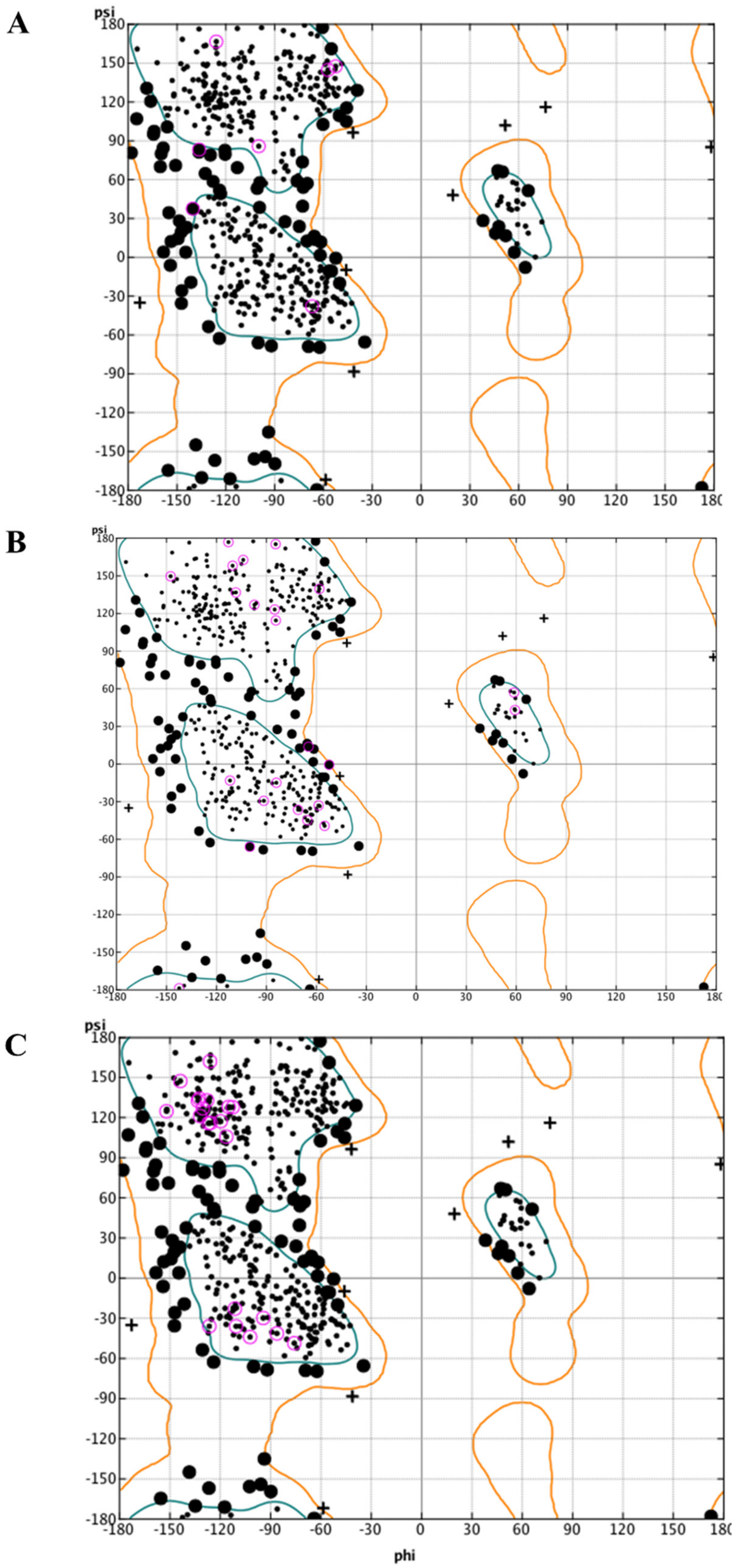

Figure S6. Ramachandran plot of epitopes. Residues of (A) Ctl, (B) EP1, and (C) EP2 are highlighted in magenta circles in different quadrants of the plot.

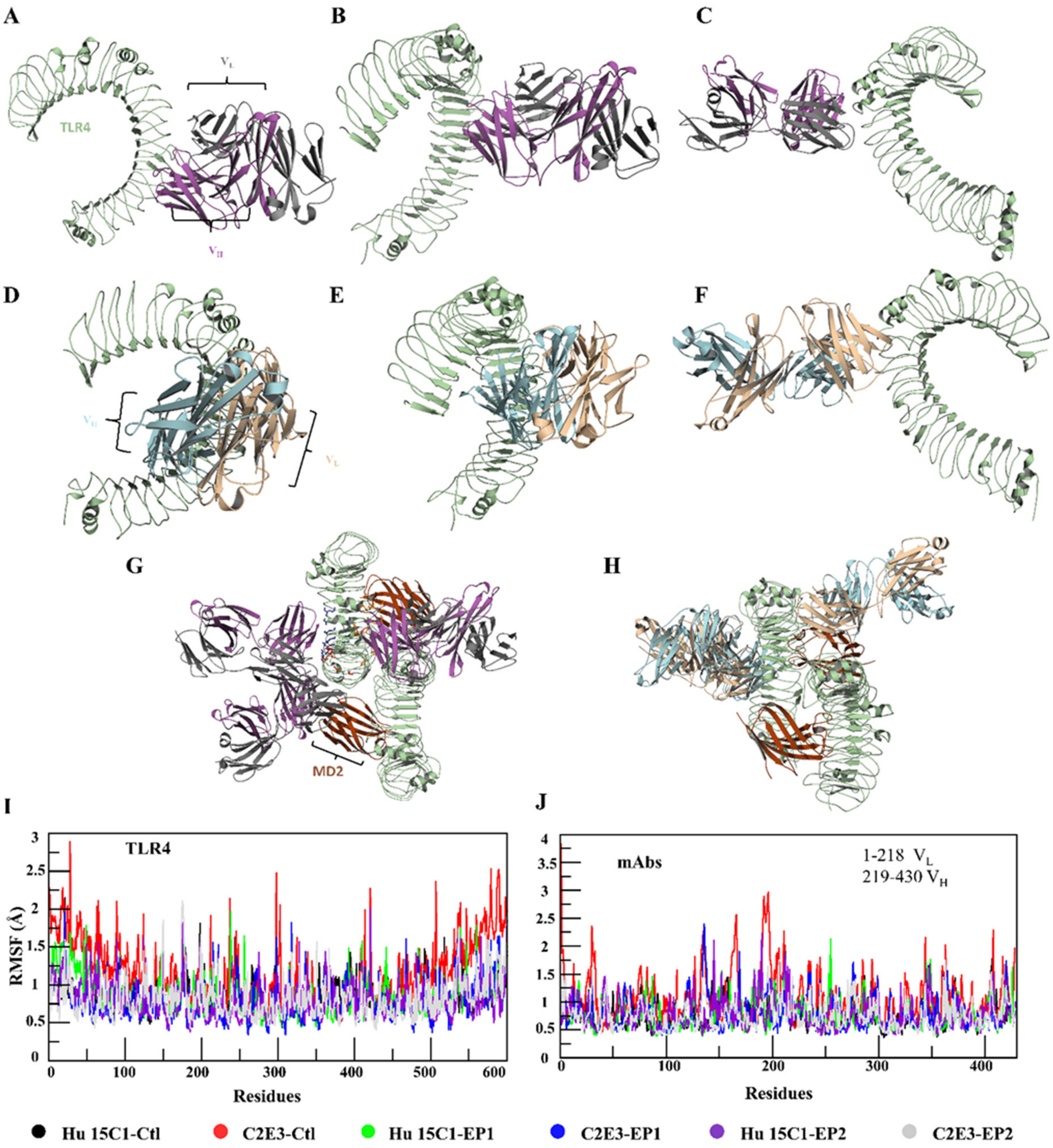

Figure S7. Hu 15C1 and C2E3 prevent the dimerization of human TLR4 by binding at different sites. Hu 15C1 and C2E3 Fab were docked to TLR4 at three epitopes: (A, D) Ctl, (B, E) EP1, and (C, F) EP2. Superimposition of the structure of the human TLR4-MD2 dimer: the first TLR4 monomer with (G) Hu 15C1 Fab, and the second TLR4 monomer with (H) C2E3 Fab. RMSF of (I) TLR4's and (J) antibodies' residues during complex formation.

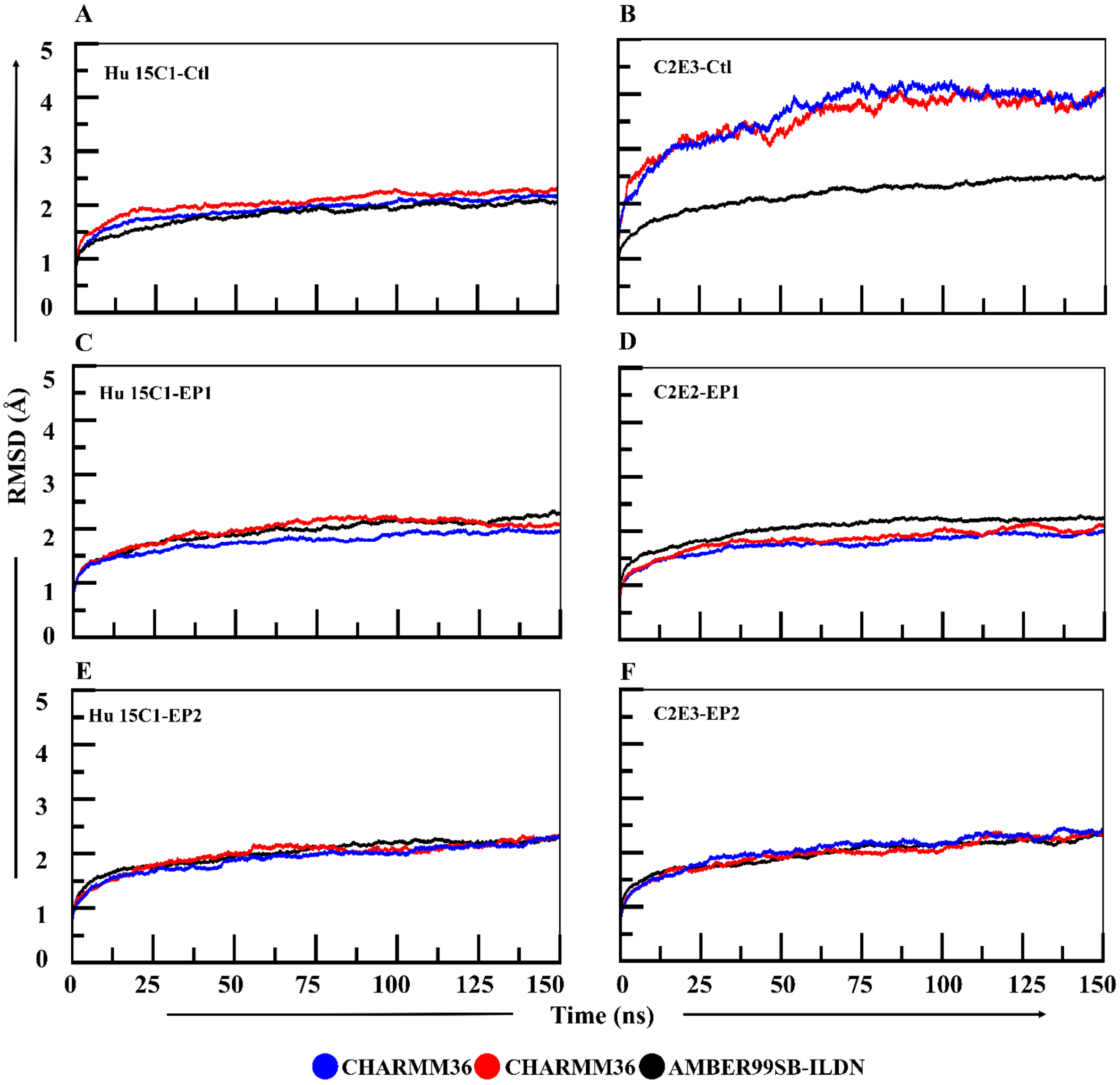

Figure S8. RMSD of TLR4-mAbs at three sites. The change in the RMSD at three epitopes: (A, B) Ctl, (C, D) EP1, and (E, F) EP2 simulated with CHARMM36 (blue and red) and AMBER99SB-ILDN (black).

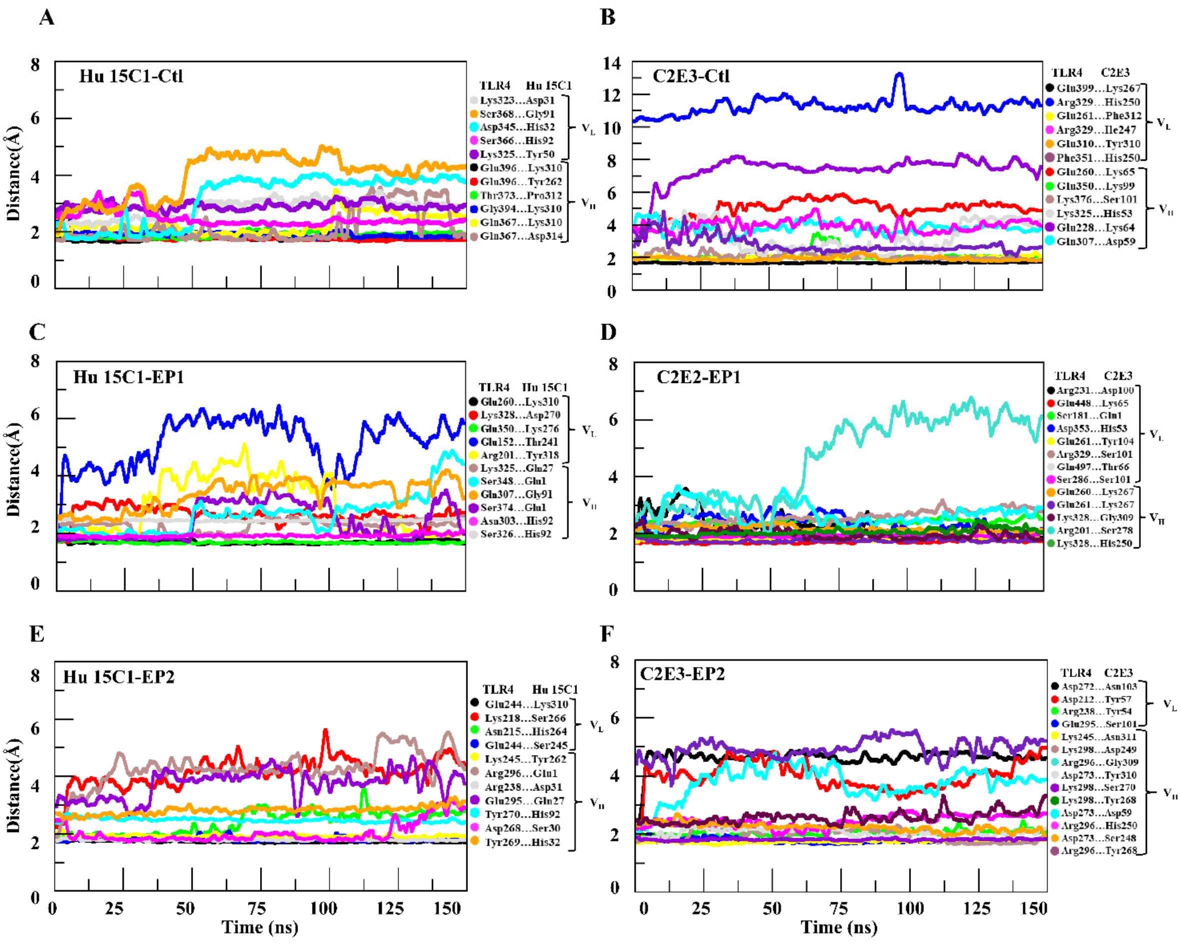

Figure S9. Interface analysis. The minimum distances of Hu 15C1 and C2E3 to TLR4's interacting residues in epitopes (A, B) Ctl, (C, D) EP1, and (E, F) EP2.

Table S1. The residues of Hu 15C1′s and C2E3′s interfaces with TLR4 at reported epitope (Ctl).

Reported Epitope (Ctl)							
**mAb**		**TLR4**	**Fab**	**Bond**	**$E_{kcal/mol}$**	**Dist. Å**	**Frequency**
**Hu 15C1**	V_L	Lys323	Asp31	IH	-29.4	2.86	4
		Ser368	Gly91	H	-2.5	2.79	1
		Asp345	His32	H	-2.1	3.02	1
		Ser366	His92	H	-1.6	3.37	1
		Lys325	Tyr50	A	-1.6	3.59	1
	V_H	Glu396	Lys310	IH	-24.54	2.76	3
		Glu396	Tyr262	H	-5.1	2.6	1
		Thr373	Pro312	H	-4.7	2.83	1
		Gly394	Lys310	H	-3.6	2.98	1
**C2E3**	V_H	Glu260	Lys65	IH	-26.88	2.82	3
		Glu350	Lys99	IH	-23.94	3.08	3
		Lys376	Ser101	H	-5.8	2.92	1
		Lys325	His53	H	-3.4	2.95	1
		Glu228	Leu64	H	-2.9	2.9	1
		Gln307	Asp59	H	-2.8	2.89	1
	V_L	Glu399	Lys267	IH	-31.34	2.8	4
		Arg329	His250	H	-13.5	2.93	2
		Glu261	Phe312	H	-5.9	2.75	1
		Arg329	Ile247	H	-2.7	3.1	1
		Glu310	Tyr310	H	-2.3	2.7	1
		Phe351	His250	A	0	3.66	1

Table S2. The residues of Hu 15C1′s and C2E3′s interfaces with TLR4 at EP1.

**Epitope 1(EP1)**							
**mAb**		**TLR4**	**Fab**	**Bond**	**$E_{kcal/mol}$**	**Dist. Å**	**Frequency**
**Hu 15C1**	V_H	Lys325	Gln27	IH	-9.4	2.57	1
		Ser348	Glu1	H	-6	3.03	1
		Gln307	Gly91	H	-3.4	2.79	1
		Ser374	Glu1	H	-3.1	2.64	1
		Asn303	His92	H	-2.4	2.91	1
		Asn303	Ser30	H	-1.2	2.97	1
		Ser348	Ser93	H	-0.9	3.06	1
		Ser326	His92	H	-0.7	3.65	1
		Asn303	Asp59	H	-0.6	3.32	1
	V_L	Glu260	Lys310	HI	-26.64	2.8	3
		Lys328	Asp270	HI	-23.41	2.77	3
		Glu350	Lys276	HI	-18.45	2.98	3
		Glu152	Thr241	H	-2.2	2.87	1
		Arg201	Tyr318	H	-2.2	2.97	1
		Glu261	Tyr265	A	-1.1	3.13	1
		Ser286	Tyr265	H	-0.7	2.93	1
**C2E3**	V_L	Arg231	Asp100	IH	-25.99	3.07	7
		Glu448	Lys65	IH	-19.91	2.66	2
		Ser181	Gln1	H	-5.9	2.75	1
		Asp353	His53	H	-5.3	2.77	1
		Glu261	Tyr104	H	-2.7	2.72	1
		Arg329	Ser101	H	-2.4	2.9	1
		Gln497	Thr66	H	-1.9	3.18	1
		Ser286	Ser101	H	-1.7	2.64	1
		Phe351	His53	A	-0.5	4.05	1
		His400	Tyr57	A	0	3.94	1
	V_H	Glu260	Lys267	IH	-25.14	2.73	2
		Glu261	Lys267	IH	-12.91	2.94	2
		Lys328	Gly309	H	-7.2	3	1
		Arg201	Ser278	H	-3.7	2.92	1
		Lys328	His250	H	-2.4	2.87	1
		Glu228	Ser274	H	-1.1	3.32	1

Table S3. The residues of Hu 15C1′s and C2E3′s interfaces with TLR4 at EP2.

Epitope 2(EP2)							
mAb		TLR4	Fab	Bond	$E_{kcal/mol}$	Dist Å	Frequency
Hu 15C1	V_H	Arg296	Glu1	IH	-25.95	3.09	6
		Arg238	Asp31	IH	-20.7	3.05	4
		Glu295	Gln27	H	-6.5	2.88	1
		Tyr270	His92	H	-3.7	3.05	1
		Asp268	Ser30	H	-1.6	2.53	1
		Tyr269	His32	H	-0.7	3.93	1
	V_L	Glu244	Lys310	H	-8.2	2.85	1
		Lys218	Ser266	H	-7.2	2.87	1
		Asn215	His264	H	-5.8	3	1
		Glu244	Ser245	H	-4.6	2.62	1
		Lys245	Tyr262	H	-1.8	2.98	1
C2E3	V_L	Asp272	Asn103	H	-3.9	2.7	1
		Asp212	Tyr57	H	-3.3	2.61	1
		Arg238	Tyr54	H	-2.3	3.01	1
		Glu295	Ser101	H	-1.7	2.89	1
		Glu244	Tyr51	H	-1.2	2.95	1
		Glu240	Ser55	H	-1	2.74	1
	V_H	Lys245	Asp311	IH	-23.13	2.85	2
		Lys298	Asp249	IH	-16.21	2.99	4
		Lys298	Tyr268	H	-7.8	2.65	1
		Arg296	Gly309	H	-6.6	2.78	1
		Lys298	Ser270	H	-6.6	2.92	1
		Lys248	Ser246	H	-5.8	3.02	1
		Asp273	Tyr310	H	-5.1	2.59	1
		Arg296	His250	H	-4.9	2.76	1
		Asp273	Ser248	H	-2.4	2.81	1
		Arg296	Tyr268	A	-0.5	4.58	1
